# Molluscan Genomes Reveal Extensive Differences in Photopigment Evolution Across the Phylum

**DOI:** 10.1093/molbev/msad263

**Published:** 2023-12-01

**Authors:** Kyle E McElroy, Jorge A Audino, Jeanne M Serb

**Affiliations:** Ecology, Evolutionary, and Organismal Biology, Iowa State University, Ames, IA, USA; Ecology, Evolutionary, and Organismal Biology, Iowa State University, Ames, IA, USA; Department of Zoology, University of São Paulo, São Paulo, Brazil; Ecology, Evolutionary, and Organismal Biology, Iowa State University, Ames, IA, USA

**Keywords:** opsins, evolution, mollusks, cryptochromes

## Abstract

In animals, opsins and cryptochromes are major protein families that transduce light signals when bound to light-absorbing chromophores. Opsins are involved in various light-dependent processes, like vision, and have been co-opted for light-independent sensory modalities. Cryptochromes are important photoreceptors in animals, generally regulating circadian rhythm, they belong to a larger protein family with photolyases, which repair UV-induced DNA damage. Mollusks are great animals to explore questions about light sensing as eyes have evolved multiple times across, and within, taxonomic classes. We used molluscan genome assemblies from 80 species to predict protein sequences and examine gene family evolution using phylogenetic approaches. We found extensive opsin family expansion and contraction, particularly in bivalve xenopsins and gastropod G_o_-opsins, while other opsins, like retinochrome, rarely duplicate. Bivalve and gastropod lineages exhibit fluctuations in opsin repertoire, with cephalopods having the fewest number of opsins and loss of at least 2 major opsin types. Interestingly, opsin expansions are not limited to eyed species, and the highest opsin content was seen in eyeless bivalves. The dynamic nature of opsin evolution is quite contrary to the general lack of diversification in mollusk cryptochromes, though some taxa, including cephalopods and terrestrial gastropods, have reduced repertoires of both protein families. We also found complete loss of opsins and cryptochromes in multiple, but not all, deep-sea species. These results help set the stage for connecting genomic changes, including opsin family expansion and contraction, with differences in environmental, and biological features across Mollusca.

## Introduction

Light is an important cue for many biological processes. Generalized photoreception is used for food production, entraining circadian rhythm, setting of photoperiodism, and regulating physiological processes, such as vasorelaxation and gonadal growth (Bradshaw and Holzapfel 2007; Nakane et al. 2010; Sikka et al. 2014; Yu and Fischer 2019; Hu et al. 2022). In animals, proteins from 2 major gene families bind to light-absorbing chromophores to form photopigments that transduce light signals: opsin and cryptochrome (CRYs). Opsin, a type of G-protein coupled receptor (GPCR) that forms a photopigment with retinal, can absorb light at specific wavelengths across the UV-visible spectrum into near infrared. Though opsins are a diverse family of GPCRs they have a highly conserved 7-transmembrane (TM) structure and lysine residue necessary for retinal binding, homologous to K296 in Bovine Rhodopsin (reviewed in Smith 2010). Opsins are classified based on the type of photoreceptors they were discovered in (e.g. rhabdomeric “r-opsins” and ciliary “c-opsins”), the G-protein they couple with (e.g. G_q_ vs. G_t_), and phylogenetic relationship (e.g. “tetraopsins”) (Shichida and Matsuyama 2009; Porter et al. 2011). The most well-studied opsins are those used in vision (referred to as “visual opsins”), such as c- and r-opsins, which are densely packed in membrane specializations of photoreceptor cells (Nilsson 2021). But opsin proteins are not just found in the eyes. Opsins are expressed in dermal melanophores and chromatophores (Ramirez and Oakley 2015), light organs (Tong et al. 2009), brains, and adrenal glands (Ohuchi et al. 2012) (Table 1). While most opsins are used for light-dependent functions, members of the opsin family have been co-opted for light-independent sensory modalities including taste (Leung et al. 2020), auditory (Senthilan et al. 2012), mechanoreception (Katana et al. 2019), and temperature reception (Shen et al. 2011) (reviewed in Leung and Montell 2017). Therefore, opsins are a remarkably diverse GPCR group with a myriad of sensory functions.

**Table 1:** Summary of key information associated with opsin types and current unknowns

Opsin type	G-protein	Signaling cascade	Cell response	Stability	Photoreceptor cell type	Functions
r-opsin (canonical)	G_q_	Phosphoinositol	Depolarize	Bistable	Rhabdomeric	Visual opsin mainly expressed in the eyes of protostomes, but also found in extraocular tissues and in some deuterostomes. Involved in phototaxis, circadian clock, and photoentrainment.
r-opsin (noncanonical)	Likely G_q_	Unknown	Unknown	Unknown	Nonvisual cells but largely unknown	Functions largely unknown, but evidence of expression in nonvisual cells of planarians suggests a role in phototaxis.
c-opsin	G_t_/G_i_	Cyclic nucleotide	Hyperpolarize	Monostable	Ciliary	Visual opsin mainly expressed in the eyes of vertebrates, but also found in extraocular tissues and in some invertebrates.
Xenopsin	G_i_	Cyclic nucleotide	Unknown^a^	Bistable	Ciliary/Rhabdomeric	Poorly characterized with unknown functions in protostomes.
Cnidopsin	G_s_/G_q_/G_c_	Cyclic nucleotide	Depolarize	Monostable	Ciliary and nonvisual cells	Visual opsin in cnidarian ocular organs, also mediates light-dependent reproductive control.
Retinochrome	Not coupled to G-proteins	Unknown	Unknown	Bistable	Rhabdomeric	Photoisomerization of all-*tran*s retinal to the 11-*cis* form in mollusks. Function poorly characterized in other invertebrates.
RPE-retinal G-protein receptor (RGR)	Likely not coupled to G-proteins	Unknown	Unknown	Bistable	Pigment cells	Photoisomerization of all-*tran*s retinal to the 11-*cis* form in vertebrates. Function poorly characterized in invertebrates.
Peropsin	G_i_/G_s_	Cyclic nucleotide	Unknown	Bistable	Visual and nonvisual cells	Likely function includes light-dependent regulation of retinal cycling in vertebrates and invertebrates, however not characterized yet.
Go-opsin	G_o_	Cyclic nucleotide	Hyperpolarize	Bistable	Ciliary/Rhabdomeric	Function poorly characterized with expression in ocular tissues of invertebrates.
Neuropsin (opsin-5)	G_i_	Cyclic nucleotide	Hyperpolarize	Bistable	Ciliary and nonvisual cells	Light-dependent functions, including circadian photoentrainment, in ocular and extraocular tissues of vertebrates. Function poorly characterized in invertebrates.

References in supplementary table S1, Supplementary Material online

See also Fig. 1 in Vöcking et al. (2022) for additional details.

^a^Cell response is likely depolarization.

Even though visual opsins have been largely characterized in some animal lineages, such as vertebrates, we know little about opsin expression, function, and evolution in invertebrates, particularly for nonvisual opsins. Mollusks are a great group of animals to explore questions about the evolution of light sensing. Eyes have evolved multiple times across, and within, taxonomic classes (Serb and Eernisse 2008; Audino et al. 2020). These organs represent a range of eye complexity, from a simple pit eye to elaborate structures such as the compound eyes analogous to those in arthropods, to the camera-type eyes, and eyes with mirror-based optics. Light sensing structures also vary across ontogeny, where most molluscan larvae have a pair of simple eyespots that are lost during metamorphosis (Raven 1966). Even without eyes, nearly all adult mollusks have a photosensitive mantle (Kennedy 1960), the membranous organ that lines both shell valves (Kennedy 1960; Carter et al. 2012). In gastropods and bivalves, these epithelial tissues of the mantle can develop into sensory extensions as papillate, lobes, or tentacles that perform a variety of modalities, including chemo-, photo-, and mechano-sense (e.g. Audino and Marian 2016; Audino and Marian 2020). Likely, the ancestral mollusk had 7 types of opsins: canonical r-opsins, noncanonical r-opsins, xenopsins, retinochromes, G_o_-opsins, and neuropsins (Ramirez et al. 2016) (Table 1). However, outside of the canonical r-opsins used for vision, little is known about the function or spatial expression of these other opsin clades (but see Kingston et al. 2015).

In contrast to opsins, the second photopigment-forming protein family, the cryptochromes, are restricted to blue-light sensitivity. Cryptochromes are a class of flavoproteins that bind to a flavin cofactor, flavin adenine dinucleotide (FAD). Cryptochromes primarily entrain the circadian clock and are highly conserved at the level of sequence and function (reviewed in Öztürk et al. 2007; Öztürk 2017). Cryptochromes belong to a larger protein family with the photolyases (PLs) that share sensitivity to blue light and are found ubiquitously across cellular life (Lin and Todo 2005; Mei and Dvornyk 2015; Öztürk 2017). While PLs repair UV-damaged DNA, cryptochromes have evolved other light directed-functions in plants and animals (see Table 2 for summary of CRY-photolyase (PL) functions). Regardless of taxonomic lineage or habitat, all cryptochrome-based photopigments absorb light in the near UV to blue range (λ_max_ between 370–440 nm; Sancar 2000).

**Table 2:** Summary of taxonomic distribution and known key functions for cryptochrome (CRY)/ PL protein families

CRY-PL family	Taxa	Functions
CPD-I	M, F, P, E, B, A	UV-light induced CPD DNA-damage repair
CPD-II (phr)	M, F, P, E, B, A	UV-light induced CPD DNA-damage repair
CPD-III	M, P, E, B, A	UV-light induced CPD DNA-damage repair
PCRY	P	Light-sensitive plant cryptochrome, involved with circadian clock regulation and various photomorphogenic roles
PCRY-like	M, P, E	Largely unknown
CRY-DASH	M, F, P, E, B, A	UV-light induced ssDNA-damage repair. Also has circadian clock functions; regarded as “intermediate” or “link” between DNA-damage repair PLs and cryptochromes
PPL	P, E	UV-light induced CPD DNA-damage repair
DCRY (CRY-I)	M	Light-sensitive animal cryptochrome. Photoreceptor activated by blue light, resets circadian clock.
CRY4	M	Magnetoreception in migratory vertebrates
6-4 PL	M, F, P, E, B, A	UV-light induced 6-4 DNA-damage repair
MCRY (CRY-II)	M	Light-insensitive animal cryptochrome, involved with circadian clock regulation.

References in supplementary table S2, Supplementary Material online.

Metazoa (M), Fungi (F), Viridiplantae (P), other unicellular eukaryotes (E), Bacteria (B), Archaea (A), pyrimidine-pyrimodone (6-4), cyclobutane-pyrimidine dimers (CPD), single-stranded DNA (ssDNA).

Here, we conducted a phylum-wide characterization of opsin and CRY-PL genomic content based on de novo gene prediction for these 2 protein families. We use these data to explore how different photopigment gene families evolve in this diverse phylum. Recent work characterized opsin genomic content to describe ancestral states for opsin number in different mollusk classes and contrast the rich molluscan opsin repertoire to other, opsin-poor, lophotrochozoan phyla, like Platyhelminthes (De Vivo et al. 2023). Our work adds to these findings by sampling more molluscan taxa (80 vs. 22 species), enabling a deep account of cross-taxa differences and similarities in opsin evolution. Furthermore, we evaluated differences in evolutionary patterns across opsin clades, which may provide insight into functional properties of the poorly understood opsin groups common to mollusks. Using a phylogenetic framework, we were able to identify dramatic differences in evolutionary dynamics within opsins and between 2 light-sensing gene families (opsin vs. cryptochrome). Several of the opsin gene lineages repeatedly were duplicated in a taxon-specific manner and were in stark contrast to other genes that were evolutionarily static. Because our results only reflect genomic presence, with many sequences identified as fragments, we stress that our findings do not have direct functional interpretations and instead should be viewed as a broad description of opsin and cryptochrome genomic content in mollusks that we hope will prompt future investigation into the causes for the huge variation in opsin abundance we found across Mollusca.

## Materials and Methods

We downloaded genome assemblies for 83 molluscan species from NCBI GenBank (where available, see supplementary table S3, Supplementary Material online for exceptions), including 39 bivalves, 8 cephalopods, 35 gastropods, and 1 polyplacophoran—the chiton, *Acanthopleura granulata*. We collected, as summary statistics for these genome assemblies, the number of scaffolds, scaffold N50, and total assembly sequence length using bbmap version 37.36 statswrapper.sh (https://github.com/BioInfoTools/BBMap) (supplementary table S3, Supplementary Material online).

### Molluscan Species Tree

To create a phylogenetic framework to examine opsin and cryptochrome evolution, we generated a molluscan species tree based on the genome assemblies. We identified orthologs for tree inference using the Benchmarking Universal Single-Copy Orthologs (BUSCO) software (Manni et al. 2021). The BUSCO single-copy genes exhibit high sequence conservation and, with nearly 1,000 sequences, represent a large dataset. We ran BUSCO (version v5.2.2) using the obd10 metazoan and molluscan databases on each of the 83 assemblies. We found that the “complete” scores were generally higher with the metazoan database compared with the molluscan database (supplementary fig. S1, Supplementary Material online) and therefore used the metazoan results for our phylogenetic analysis. To remove assemblies of low quality for gene discovery, we plotted (R version 3.6.2; ggplot2 version 3.3.5) the BUSCO % complete scores as a histogram to determine natural breaks in results (supplementary fig. S1, Supplementary Material online). Three assemblies less than 73% complete were removed from subsequent analyses (*Limnoperna fortunei*: GCA_003130415.1; *Panopea generosa*: GCA_902825435.1; *Pinna nobilis*: GCA_016161895.1).

From the remaining 80 genomes (1 polyplacophoran, 8 cephalopods, 35 cephalopods, and 36 bivalves), we found 899 complete single copy BUSCO genes present in at least 75% (60 out of 80) genomes and these were used to estimate the species tree. Genes were aligned with MAFFT (version 7.453; Kuraku et al. 2013) using the “auto” parameter. Alignments were quality trimmed using trimal (version 1.4.rev22; Capella-Gutiérrez et al. 2009) with the “-automated1” parameter. We ran IQ-TREE2 (version 2.1.3; Minh et al. 2020) on all 899 protein sequence alignments to search for the best-fitting model of protein sequence evolution for each gene with ModelFinder (Kalyaanamoorthy et al. 2017). We then used catsequences (https://zenodo.org/record/4409153#.Y8gnmXbMJPY) to concatenate the 899 trimmed amino acid alignments and combined the accompanying partition configuration file with the ModelFinder results as input for the partitioned analysis in IQ-TREE2 (Chernomor et al. 2016). Branch support of the maximum-likelihood results was determined with 1,000 replicates for each: ultrafast bootstrap, SH approximate likelihood ratio test, and an approximate Bayes test (parameter: –alrt 1000 -B 1000 –abayes) (Anisimova et al. 2011; Hoang et al. 2018).

### Reference Opsin set for Molluscan Gene Annotation

Genome annotation strategy and quality may differ widely across genomes, which can lead to the false appearance of “lineage-specific” genes (Weisman et al. 2022), thus, weakening comparative genomic analyses like gene family evolution. Furthermore, many genome assemblies lack publicly available annotations entirely, including 18 of the 80 genomes analyzed here. For these reasons, we opted for de novo prediction of opsin and CRY-PL genes to have a consistent process in generating datasets (i.e. protein sequences) for phylogenetic analysis. We applied the targeted gene annotator pipeline BITACORA (version 1.3; Vizueta et al. 2020), which incorporates the homology-based gene predictor, Gene Model Mapper (GeMoMa) (Keilwagen et al. 2016, 2018) and leverages its parameter for annotating specific genes (vs. genome-wide annotation) to predict opsin sequences. Because BITACORA uses a reference set of protein sequences and Hidden Markov Models (HMMs) as queries in tblastn and hmmer searches, high-quality input data is needed for accurate gene prediction. We developed a high-quality reference set of molluscan opsin sequences from 9 molluscan species based on the following criteria: (i) identification of 7 TM domains, (ii) presence of lysine residue for chromophore binding, and (iii) complete coding sequence. Protein sequences were downloaded from genome annotation of 9 mollusk species representing a diverse sampling of molluscan lineages (1 cephalopod, 4 gastropods, and 4 bivalves; bolded in supplementary table S3, Supplementary Material online). We used blastp (BLAST+ version 2.6.0; Camacho et al. 2009) with an e-value of 1e-20 to query opsins from the metazoan-wide opsin gene collection in Ramirez et al. (2016) against each of 9 molluscan protein sets. Next, we used HMMER version 3 .1b1 hmmscan (hmmer.org) to search the Ramirez et al. (2016) hits against the protein families (Pfam) database version 35.0 (Mistry et al. 2021) and retained sequences with hits to the 7tm_1 (PF00001.24) domain “7-TM receptor (rhodopsin family).” We then visually screened sequences for the conserved lysine residue (K296 in Bovine rhodopsin) necessary for retinal binding in MEGA X (Kumar et al. 2018). Finally, we manually curated gene models using GeneWise (Birney et al. 2004; Madeira et al. 2019) based on tblastn hit coordinates in each species’ genome assembly. For all 9 species, we ensured that start and stop codons were present in an open reading frame of concatenated exons.

We found that the protein names from public databases NCBI and PROSITE were often not specific enough (e.g. “rhodopsin-like”) for our purposes and several of the species’ datasets lacked functional annotations. Therefore, we phylogenetically analyzed this panel of molluscan opsins to assign specific clade-level names so that we could use these sequences downstream in our classification of de novo predicted opsin sequences. To classify the opsin sequences in our molluscan reference panel, we used MAFFT to align these putative opsin sequences to known molluscan opsins (Ramirez et al. 2016) and the outgroup sequences used in Vöcking et al. (2017), which includes opsin-like sequences from the placozoan, *Trichoplax adhaerens*, known as “placopsins,” melatonin receptors, along with other GPCRs such as adrenergic, dopamine, and octopamine receptors (also used in Döring et al. 2020). Next, the sequence alignment was manually trimmed, and a maximum likelihood tree was generated with IQ-TREE2 (−alrt 1000 -B 1000 –abayes). We then assigned the following classifications to each sequence in our reference set based on their phylogenetic placement: r-opsin, nonconical r-opsin, xenopsin, retinochrome/RGR/peropsin, G_o_-opsin, neuropsin. We added to the reference set 2 neuropsin sequences from the slug *Ambigolimax valentianus* and a xenopsin from the squid *Idiosepius paradoxus* to complement missing/truncated sequences from heterobranch gastropods and cephalopods (see supplementary table S4, Supplementary Material online for list of sequences in opsin reference set).

We were able to classify 107 of the 109 molluscan opsin reference sequences as canonical and noncanonical r-opsins (n = 11, n = 17, respectively), neuropsin (n = 12), G_o_-opsin (n = 11), xenopsin (n = 40), peropsin (n = 5), and retinochrome (n = 10) types (supplementary fig. S2 and supplementary table S4, Supplementary Material online). The phylogenetic distinction of “noncanonical” vs. “canonical” r-opsin follows Ramirez et al. (2016). All opsin type clades had high support values and a topology similar to previous studies (e.g. (Rawlinson et al. 2019). Unlike Vöcking et al. (2017) and Döring et al. (2020), in our results, the outgroup sequences form a strongly supported monophyletic clade and included placopsins, versus the placopsins as the sister lineage to opsins, then melatonin receptors and the other GPCRs further outside. The xenopsins were divided into 2 major clades that we assigned “a” and “b” based on the literature (supplementary fig. S2, Supplementary Material online) (Rawlinson et al. 2019; Döring et al. 2020). We also found that a group of 7 xenopsins from the bivalve *Sinovacula constricta* formed a distinct clade outside the “a” and “b” groups (90.1/81 branch support), which were named “Scon-opnGxS#” (supplementary fig. S2, Supplementary Material online). The 2 sequences in our reference set that did not clearly belong to a specific opsin clade were from *Lottia gigantea* (XP_009051446.1, labeled as Lgig-opnUNK) and *Sinovacula constricta* (evm.model.Chr17.756, labeled Scon-opnUNK). This reference gene set covers the 3 main classes of mollusks analyzed here and, for bivalves and gastropods, includes 4 distinct orders, and appears to account for all opsin groups found in Mollusca. Therefore, these sequences represent a powerful dataset for both homology-based opsin discovery in molluscan genomes and the subsequent phylogenetic classification of opsin sequences.

### Opsin Search in Molluscan Genome Assemblies and Phylogenetic Analysis

The reference set of molluscan opsins was used as input for BITACORA to generate opsin gene models from the 80 molluscan genome assemblies represented in our species tree. First, we generated an HMM profile of the reference opsins from an alignment of the 109 reference opsins using HMMR. Then, we used the “runBITACORA_genome_mode.sh” script to run BITACORA in genome mode (i.e. no input genome annotation) for de novo gene prediction using GeMoMa (parameter GEMOMA = T) based on tblastn results. We screened all the resulting protein predictions for the presence of a K296 retinal binding site (supplementary table S5, Supplementary Material online for BITACORA opsin gene counts).

We produced opsin phylogenies that included: (i) exclusively mollusk sequences identified in this study, along with outgroups (Vöcking et al. 2017); (ii) a more diverse panel of opsins, including opsin sequences from the light-interacting toolkit (Speiser et al. 2014) along with additional xenopsin and cnidopsin sequences from (Gühmann et al. 2022); and (iii) an extensive opsin tree that also includes chaopsin, ctenopsin, bathyopsin sequences (defined in Ramirez et al. 2016) and anthozoan specific opsins (ASO-I, ASO-II) from Gornik et al. (2020) (see supplementary table S6, Supplementary Material online for details). These additional opsin datasets were included to ensure that we were not inadvertently forcing the predicted molluscan sequences into certain groups, as our reference opsin panel included 2 “unknown” opsins after our initial molluscan-only phylogenetic analysis. Furthermore, additional nonmolluscan sequences could help distinguish divisions within opsin types such as the “a” vs. “b” clades of xenopsin, and clarify how the molluscan peropsins and retinochrome relate to each other. In each phylogenetic analysis, we aligned the amino acid sequences with MAFFT using the E-INS-i iterative refinement method and trimmed the sequences with trimal using “gappyout” mode. Finally, we generated a maximum likelihood tree with IQ-TREE2 using ModelFinder to determine the best-fit protein substitution model according to Bayesian information criterion scores (LG + F + R10 selected each time). Ultrafast bootstrap (1,000 replicates), SH approximate likelihood ratio test (1,000 replicates), and approximate Bayes test were used to evaluate branch support.

To characterize patterns of gain and loss of opsins across the molluscan phylogeny, we used GeneRax (version 2.0.4; Morel et al. 2020) to generate reconciled gene trees for each major opsin type with the BUSCO-based species tree from this study (supplementary fig. S3, Supplementary Material online). We used the UndatedDL probabilistic model for computing the reconciliation likelihood and the SPR tree search mode. We used ThirdKind to visualize the reconciliation results (Penel et al. 2022).

### Cryptochrome Identification and Phylogenetic Analysis

We employed a similar strategy for identifying cryptochrome sequences as for opsins. We queried the same 9 genome annotations for potential cryptochrome sequences by blastp hits to cryptochrome amino acid sequences from Rivera et al. (2012). From the blast hits, we ran hmmscan against the pfam database and retained sequences with FAD binding domain of DNA PL (PF03441.17) and DNA PL (PF00875.21). We then combined our hits with cryptochrome amino acid sequences used in Deppisch et al. (2022) as landmarks to classify the sequences for our molluscan reference cryptochrome set. We aligned sequences with MAFFT (LINSI), trimmed the alignment with trimal (automated1), and produced a maximum-likelihood tree with IQ-TREE2 (LG + R5). We then classified the mollusk sequences as phr (*photorepair* gene in *Drosophila*, belonging to CDP-II), CRY-DASH (*Drosophila*, *Arabidopsis*, *Synechocystis*, human cryptochromes), (6-4)PLs, CRY-I (animal cryptochrome-1, the “mammal”-type, i.e. MCRY), and CRY-II (animal cryptochrome-2, the “Drosophila”-type, i.e. DCRY) based on their phylogenetic groupings (supplementary fig. S4, Supplementary Material online). For the CRY-PL molluscan reference sequences, we classified all 43 sequences into CDP-II (n = 9), CRY-DASH (n = 8), 6-4PL (n = 6), CRY-I (n = 9), CRY-II (n = 9) from the same 9 mollusk species (summarized in supplementary table S7, Supplementary Material online). We used the 43 classified sequences as a database for BITACORA to search for cryptochromes in the rest of the 80 molluscan genome assemblies. The predicted genes from BITACORA were then screened against the pfam database with hmmscan for FAD binding and DNA PL domains (see supplementary table S5, Supplementary Material online for BITACORA CRY-PL counts). As with the opsins, we produced an initial CRY-PL tree and searched for possible “missing” sequences first with blastp against the BITACORA output in case our filtering step removed a positive CRY-PL sequence and then using tblastn and GeneWise to generate protein models (16 CRY-PL sequences total, 51 opsins). We also removed apparent contaminant sequences that grouped outside of the mollusk clades. These 4 sequences were located on short scaffolds in their respective genome assemblies and aligned to bacterial sequences via blastp. Our final CRY-PL phylogeny was produced with IQ-TREE2 (LG + R8) and included amino acid sequenced from Gornik et al. (2020), the LIT (cry2_default_clock.fas from LIT_1.1 included with PIA2 at https://github.com/MartinGuehmann/PIA2), and landmark sequences used in (Deppisch et al. 2022) to aid in broader CRY-PL classification.

### Photic Environment and Eye Type

To complement our investigation with general information on molluscan ecology and visual systems, we gathered data from the literature for all 80 species, focusing on the presence of eyes in the adult stage. Even though most mollusks have a photosensitive mantle, eyeless species are defined by the absence of a structure capable of creating images or detecting light direction. We also gathered information for eye type, optical components, environment, and aquatic habitat depth. All traits, states, and respective references are listed in supplementary table S8, Supplementary Material online.

## Results and Discussion

### Identification and Classification of Mollusk Opsins From Genome Assemblies

**Fig. 1.**
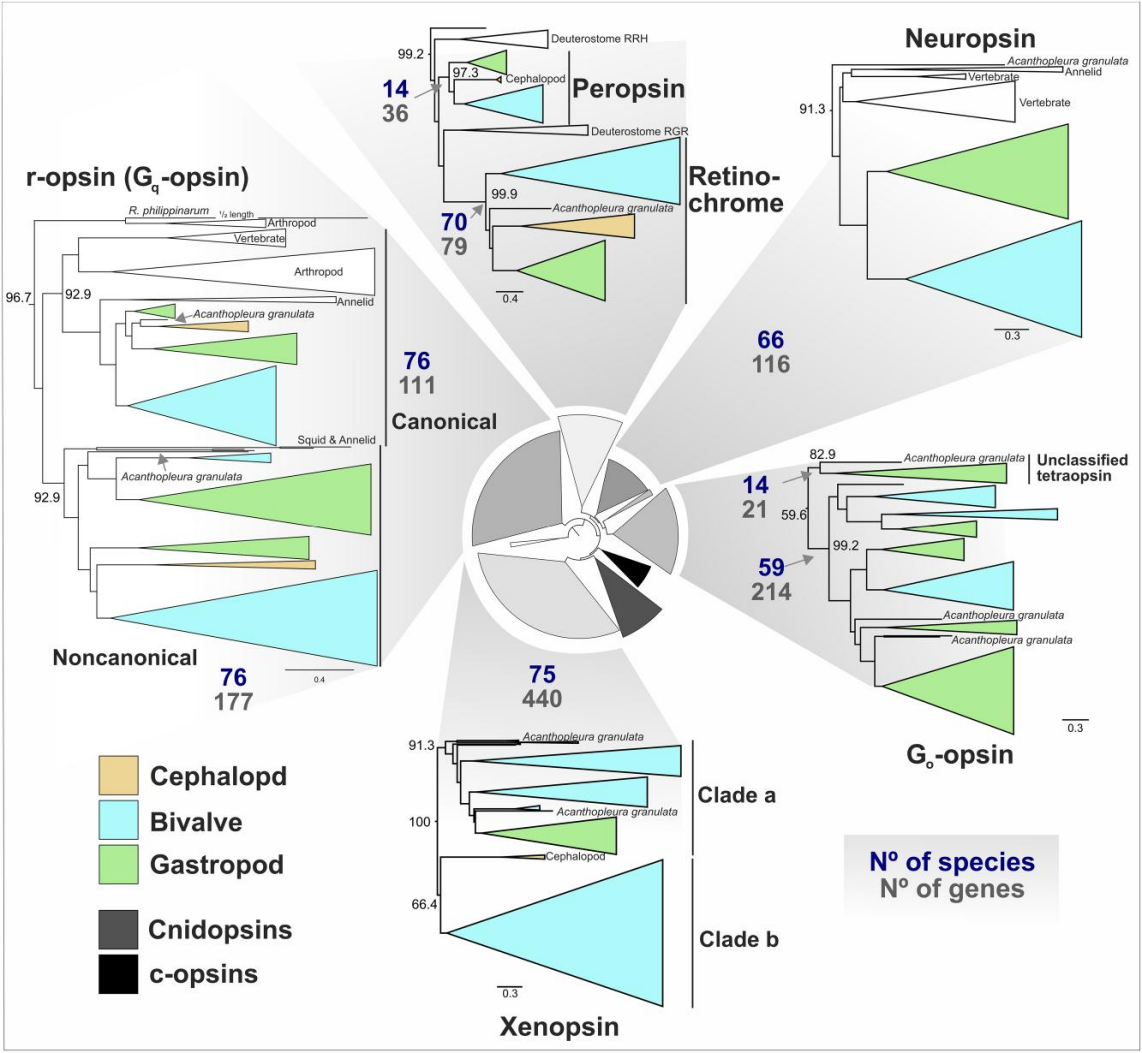
Opsin clades differ in total size and number of species represented. Subtrees of mollusk containing opsin clades displayed from the IQ-TREE2 ML opsin phylogeny (LG + F + R10 amino acid substitution), shown at center. For each of 7 opsin types found in mollusks (plus the “Unclassified tetraopsin group), the total number of sequences in the tree (N^o^ of genes) and species (N^o^ species) with that opsin are displayed. Clades are collapsed according to taxonomic clades (e.g. bivalves) and color coded by mollusk class. UF-bootstrap support values shown at base of named of opsin clades. Support values for inner circular tree are shown in supplementary fig. S12a, Supplementary Material online and mollusk-specific clades in supplementary fig. S6, Supplementary Material online; full tree with support values is in supplementary fig. S7, Supplementary Material online.

Across the phylum, mollusk genomes contain opsins from as many as 7 distinct clades but lack c-opsins and cnidopsins (Fig. 1). We were able to phylogenetically place 1,174 out of 1,196 predicted opsin sequences into 1 of these 7 opsin groups (canonical or noncanonical r-opsins, neuropsin, G_o_-opsin, xenopsin, peropsin, and retinochrome) with high support values (supplementary fig. S5, Supplementary Material online). These classifications held whether we produced a mollusk-only tree (supplementary fig. S5, Supplementary Material online) or included sequences from other taxa (e.g. deuterostome, arthropod, annelid RGR/peropsins; details in supplementary table S9, Supplementary Material online) (supplementary fig. S6, Supplementary Material online). In fact, the phylogeny that also included c-opsins, cnidopsins, and nonmolluscan xenopsins, had clearer separation of “a” and “b” xenopsin subclades (e.g. as seen in Rawlinson et al. 2019). In the mollusk-only tree (supplementary fig. S5, Supplementary Material online), a group of sequences from heteroconch bivalves forms an additional xenopsin clade like the sequences from *Sinovacula constricta* in the reference opsin panel (supplementary fig. S2, Supplementary Material online). Those heteroconch-specific sequences fall under clade “b” in the more diverse opsin tree (see “Scon-opnGxS” sequences in supplementary fig. S7, Supplementary Material online). Our findings do not reveal any other major groups of opsins in mollusks that were not already identified from gene expression data (Ramirez et al. 2016).

Of the remaining 22 gene sequences that were not phylogenetically placed into 1 of the 7 main opsin classes, 20 sequences formed a distinct clade of “unclassified tetraopsins” (Fig. 1, supplementary fig. S4, Supplementary Material online). The unclassified tetraopsins are typically single copy, restricted to gastropods and the lone chiton representative (Fig. 1), and are the sister group to either neuropsin (supplementary fig. S5, Supplementary Material online), G_o_-opsin (supplementary figs. S6, and S8 Supplementary Material online), or G_o_-opsin + neuropsin (supplementary fig. S9, Supplementary Material online). So, it is not clear whether this clade of opsins belongs to neuropsin, G_o_-opsin, or forms its own group outside of them. Given that no nonmolluscan sequences in our extended phylogenies grouped with these 22 sequences it seems more likely to neuropsin or G_o_-opsin, as opposed to a clade that split before the neuropsin-G_o_ split. The 2 remaining unclassified opsins are from the chiton *Acanthopleura granulata* (supplementary fig. S5, Supplementary Material online).

**Fig. 2.**
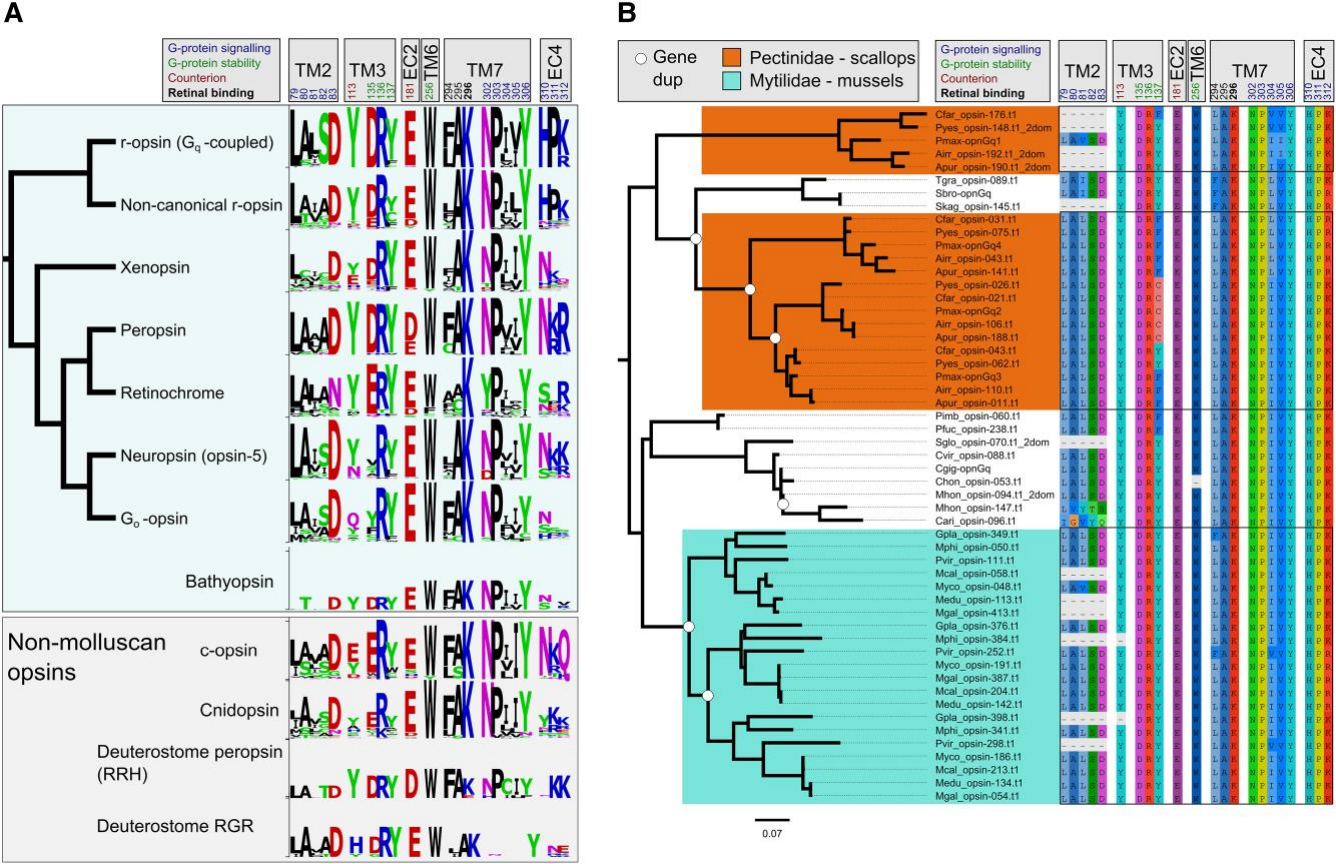
Important amino acid sites for opsin structure and function. a) Molluscan opsins sequence logos for important motifs. Logos generated with WebLogo3 (Schneider and Stephens 1990; Crooks et al. 2004). Tree of molluscan opsin relationships based on Fig. 1, supplementary figs. S5 and S6, Supplementary Material online. Additional nonmolluscan opsin logos generated from sequences used in supplementary fig. S6, Supplementary Material online and bathyopsins in supplementary figs. S7 and S8, Supplementary Material online. Heights reflect ranked abundance of amino acids at each site. b) Duplications in Pectinidae and Mytilidae canonical r-opsins (G_q_-coupled) differ in E/DRY motif. Pteriomorphia portion of GeneRax reconciled canonical r-opsin tree. Orange and teal shading and boxes highlight duplication in Pectinidae and Mytilidae, respectively. The E/DRY motif in TM3 varies across G_q_-opsin duplicates in Pectinidae [DRF (9/20), DRY (6/20), DRC (5/20)] vs. DRY in all (21/21) Mytilidae G_q_-opsins, and nearly every other molluscan G_q_-opsin. The NPxxY motif in TM7 also varies more in Pectinidae [NPVVY (2/20), NPJIV (2/20), NPLVY (5/20), NPJVY (10/20)] than Mytilidae [NPJVY (2/21), NPVVY (19/21)]. In a) and b), positions included above are based on Bovine Rhodopsin (NP_001014890.1) numbering. At top are locations relative to the protein structure (TM = transmembrane, EC = extracellular loop). Color coding of residue positions reflects function to the opsin protein, broadly based on literature from GPCRs. The retinal binding site K296 and counterion positions are critical for photopigment formation.

To classify these chiton opsins, conducted a phylogenetic analysis that included the reference molluscan opsins, opsins from the previously extended phylogeny (e.g. c-opsins and cnidopsins) and added ctenophore-specific “ctenopsins”, anthozoan-specific opsins, bathyopsins and chaopsins (defined in Ramirez et al. 2016) and produced a new maximum likelihood tree (see supplementary table S9, Supplementary Material online for dataset details). We found that the 2 chiton sequences grouped with the bathyopsins (supplementary fig. S9, Supplementary Material online), a small clade reported as the sister clade to c-opsins (Ramirez et al. 2016; Vöcking et al. 2017; Rawlinson et al. 2019). This topology was consistent when the total molluscan BITACORA opsin dataset was included, as well (supplementary fig. S8, Supplementary Material online). Bathyopsins are distinct from c-opsins because they include genes from echinoderms and brachiopods, thus representing a clade that would have predated the protostome-deuterostome split (Ramirez et al. 2016). While others (De Vivo et al. 2023) have recently reported the same chiton sequences as a c-opsin, when we compared key functional motifs between these chiton sequences to c-opsins we found the tripeptide sequence in the fourth cytoplasmic loop important for specific G-protein binding varies. Vertebrate c-opsins use the tripeptide sequence NKQ, located in the fourth extracellular loop (EC4), for G_t_ binding (Marin et al. 2000). Most of the c-opsins we analyzed, including many outside of the vertebrate c-opsins, have NKQ (Fig. 2a). The 2 chiton sequences have NSR and NST, indicating that if they function as photoreceptors, it is unlikely that they drive similar phototransduction pathways, if at all, to the c-opsins. These sequences do demonstrate, though, that c-opsin-like sequences were present in early molluscan evolution and subsequently lost for most of the phylum.

**Fig. 3.**
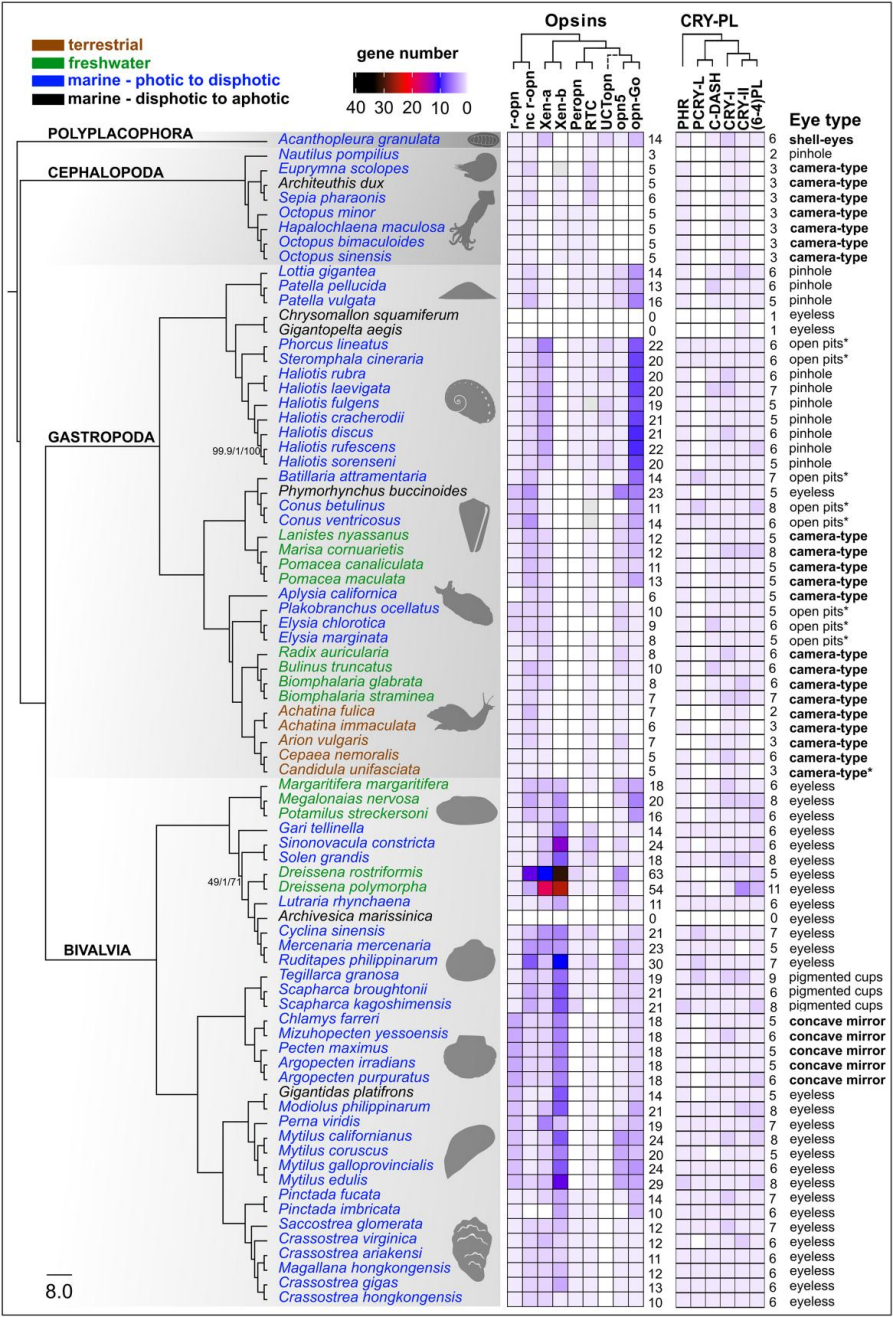
Summary of molluscan opsin and cryptochrome genomic content from 80 species. Maximum likelihood species tree generated with IQ-TREE2 based on a partitioned amino acid supermatrix from 899 complete single copy BUSCO sequences (metazoan_obd10) recovered from at least 60/80 species. Branch values are SH-aLRT % support (with 1,000 replicates)/aBayes probability/UFBoot support % (with 1,000 replicates). Branch supports shown only where any of the 3 values is less than 100%. Species tree rooted with the polyplacophoran chiton. Branches transformed with Figtree as “proportional.” Taxon names are color coded by habitat type. Opsin and cryptochrome abundances (“gene number”) reflected by heatmap. Empty squares indicate gene absence, gray likely presence. Total counts for opsins and cryptochromes included to right of each matrix. Unclassified tetraopsin indicated as “UCTopn” dashed lines indicate ambiguous phylogenetic placement. Habitat and photic environment of each species indicated by text color and eye type listed to right of figure. R script for heatmap adapted from Gornik et al. (2020). Species silhouettes obtained from PhyloPic (https://www.phylopic.org/).

While most bivalves, gastropods, and the chiton *Acanthopleura granulata* have at least 1 representative sequence for each of 7 opsin types (Fig. 1 and  3), cephalopods have the fewest number of opsins and have lost multiple opsin types. Genomes of octopus and squid cephalopods typically have 5 opsins (canonical and noncanonical r-opsins, xenopsin, and 2 sequences from the RGR/retinochrome clade), while *Nautilus* only have 3 opsins (canonical and noncanonical r-opsins, and retinochrome), indicating that neuropsin and G_o_-opsin were likely lost in the cephalopod ancestor (Fig. 1). The most opsin-rich genomes, based on our analysis, are the small freshwater *Dreissena* zebra and quagga mussels (Myida: Dreissenidiae) with 54 and 63 opsins, respectively. Such high numbers of opsins are not unprecedented, as dozens of opsins have been identified from genomes of the crustacean *Daphnia* (n = 46) (Colbourne et al. 2011) and dragonflies (n = 15 to 33) (Futahashi et al. 2015; Brandon et al. 2017).

### Dramatic Differences in Evolutionary Dynamics Across Opsin Type and Mollusk Species

To understand the broad differences in opsin type number among lineages (Fig. 3), we next examined the evolutionary dynamics of opsin gene family expansion by comparing species tree topology with and gene trees. We used the species tree derived from 899 BUSCO protein sequences. The resulting BUSCO-based species tree was largely consistent with recent molecular phylogenies (Kocot et al. 2011; Smith et al. 2011; Combosch et al. 2017; Cunha and Giribet 2019) and branch support was high (ultrafast bootstrap/approximate Bayes test/SH approximate likelihood ratio test) in all but 1 location: a bivalve clade with Adapedonta as the sister group to Myida-Venerida (49/1/71) (supplementary fig. S3, Supplementary Material online). We then generated gene trees for each major opsin type defined in Fig. 1. Species and gene trees were compared under reconciled tree analyses to characterize gain and loss patterns for each major opsin type across the molluscan phylogeny. We found that opsin types differed greatly in their size relative to one another, with some families having more of a “static” evolutionary history versus other families with “dynamic” changes, and some opsin types vary in size within particular taxonomic lineages (Table 3). In mollusks, the “static” opsin families include the canonical and noncanonical r-opsins, retinochrome, peropsin, and, to a lesser extent, neuropsin. The G_o_-opsins and xenopsins are the “dynamic” opsins families, with large changes in gene abundance across the phylum. A potential limitation to these results is that incomplete lineage sorting (ILS) can limit the performance of gene tree reconciliation analyses, including GeneRax, which can handle moderate but not high degrees of ILS (Morel et al. 2020). At present, little is known about ILS in molluscan genome evolution and evidence for broad effects of ILS in mollusks is lacking; therefore, we do not necessarily expect ILS to influence our results in a meaningful way. However, with more molluscan genomes becoming available, characterizing the extent of ILS at varying depths of divergence in mollusks will be a valuable avenue of research.

**Table 3:** Summary of “events” from GeneRax reconciliation of opsin families

Event	r-opsin	Noncanonical r-opsin	Xenopsin	Peropsin	Retinochrome	Neuropsin	Go-opsin
S	101	142	263	33	74	91	172
L	10	35	145	10	17	28	73
D	11	35	175	2	4	24	48
Total events	**102**	**142**	**293**	**25**	**61**	**87**	**147**
S%	0.90	0.80	0.60	0.94	0.95	0.79	0.78
D%	0.10	0.20	0.40	0.06	0.05	0.21	0.22

S, speciation: L, loss; D, duplication; T, total. Rows below the double line are the proportion of total events from S and D.

**Fig. 4.**
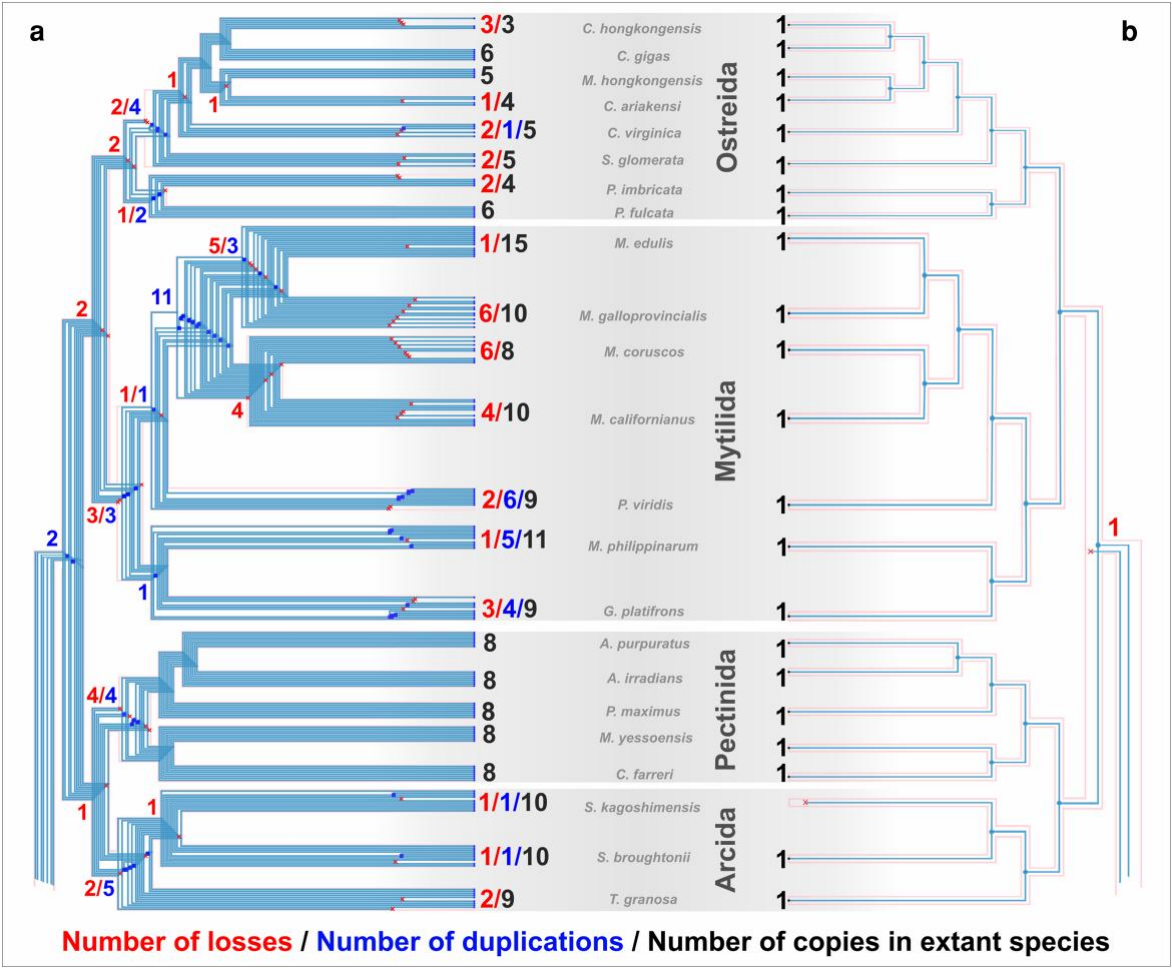
Comparison of GeneRax reconciliation for a) xenopsin and b) retinochrome evolution in Pteriomorphia. Subsets of Thirdkind visualization from opsin reconciliation with locations of gene duplication and loss given at nodes and tips of species tree along with the total observed number of genes in each species for these 2 opsins. Blue line traces evolutionary history for each gene. Xenopsin is recurrently expanded via duplication in different orders of Pteriomorphia while retinochrome remains single copy, only evolving via speciation.

The GeneRax reconciliation results indicate that most of the opsins identified from molluscan genomes are the result of speciation events rather than duplications (Table 3). In part, this result reflects the “stasis” of some opsin clades and that most duplications occur at nodes on our species tree (speciation events), as opposed to tips. Over 90% of sequences in the canonical r-opsin, peropsin, and retinochrome clades are from speciation events (Table 3). This result highlights the extreme rarity of duplications of these opsins to be retained. The noncanonical r-opsins, neuropsins, and G_o_-opsins had speciation events accounting for 78% to 80% of the sequences in our reconciled trees (Table 3). Xenopsins were by far the most dynamic group of opsins, with only 560 of the sequences in our reconciled tree coming from speciation events (Table 3). We illustrate 2 extreme cases of xenopsin vs. retinochrome from the marine bivalve species in Pteriomorphia in Fig. 4. Aside from a single loss in the blood clam, *Scapharca kagoshimensis*, retinochrome is maintained at speciation events as a single-copy gene. In contrast, xenopsin duplications and losses occur regularly throughout the Pteriomorphia (discussed below).

Lineage-specific opsin expansions have been seen in a variety of taxa, but these duplications are generally limited to one type of opsin, e.g. c-opsins in vertebrates (e.g. Borges et al. 2015, but see Beaudry et al. 2017 as relating to whole genome duplication), and r-opsins in insects (e.g. Feuda et al. 2021). Major differences in opsin content across cnidarians are accounted for by ASO-II opsins and cnidopsins, the latter being the only opsin type in Medusozoa (Gornik et al. 2020). Mollusks, due to lineage-specific expansions, exhibit huge variation for both xenopsin and G_o_-opsin abundance. While neuropsin and r-opsin tend to be more evolutionarily “static” in mollusks, we found some cases of expansion including noncanonical r-opsins in heteroconch bivalves and neuropsins in mytilids (Fig. 3). These results paint mollusks as having among the most, if not *the* most, variable opsin repertoires across the metazoan phyla.

#### Retinochrome and Peropsin are Rarely Duplicated in Mollusks

Retinochrome was among the most consistently recovered opsin, and most consistently single copy (Fig. 1, Fig. 4b). Unlike most opsins, retinochrome does not initiate phototransduction but instead functions in the visual cycle as a photoisomerase for converting all-*trans* retinal back into 11-*cis* retinal (Terakita 2005; Terakita and Nagata 2014). Originally characterized in squid (Hara and Hara 1965, 1967), retinochrome was eventually identified across Mollusca from transcriptomes (Ramirez et al. 2016) and more recently in other Lophotrocozoa phyla, including annelids and platyhelminthes (Vöcking et al. 2021; Gühmann et al. 2022). Given its critical role in the visual cycle, it is not surprising that retinochrome is rarely lost in mollusks. Also, if it primarily functions to recycle and store retinal, we might not expect much functional diversification for this opsin. In fact, we only see 2 examples of retinochrome duplication, in bivalves and squid (supplementary fig. S9, Supplementary Material online).

The bivalve duplication of retinochrome, based on GeneRax reconciliation, appears to be ancestral for the class but only retained in the heteroconch species (supplementary fig. S9, Supplementary Material online). Why a second retinochrome is retained in these bivalves is unclear and no other information exists about this duplication. A necessary first step toward understanding its biological relevance will be demonstrating if, when, and where it is expressed. The squid retinochrome duplication was first recognized in *Idiosepius* (Yoshida et al. 2015). RNA-seq from several tissues point toward 1 squid retinochrome specializing in eye expression and the other copy having a broader range, including the gut (Yoshida et al. 2015). Octopus species have a single retinochrome, but they have a peropsin, which squids lack. Given the view of retinochrome and peropsins as primarily photoisomerases (e.g. (Vöcking et al. 2021), it is tempting to consider the octopus peropsin and duplicated squid retinochrome having similar functions. Exactly how functionally similar the molluscan peropsin is to retinochrome or other peropsins (e.g. retinal pigment epithelium-derived rhodopsin homolog, “RRH” in humans) is unclear. Retinochrome and molluscan peropsin differ in key amino acids for opsin function, such as the NPxxY motif and a tripeptide important for G-protein signaling (supplementary fig. S8, Supplementary Material online). Interestingly, based on the conservation of these motifs, the molluscan peropsin may be able to drive phototransduction, as has been proposed for other peropsins but not retinochrome or RGR (Vöcking et al. 2022).

A major complication to inferring function of mollusk peropsin is that “peropsin” does not appear to be a monophyletic group, but rather a name given to several taxon-specific clades within the RGR/retinochrome group of tetraopsins: deuterostome peropsins, arthropod peropsin-like, and mollusk peropsin-like (Ramirez et al. 2016; Vöcking et al. 2021). Furthermore, phylogenies with mollusk-peropsin sequences differ in their placement (Ramirez et al. 2016; Vöcking et al. 2021; Gühmann et al. 2022) and our lack of strong statistical support for the relationships between different groups within the RGR/retinochrome/peropsin group (supplementary fig. S6 and supplementary fig. S8, Supplementary Material online) do not resolve this issue. Ultimately, our results do support retinochrome and mollusk peropsin as distinct clades and likely not sister clades within the broader RGR/retinochrome/peropsin lineage. Still, these opsins are very similar in their extreme rarity for duplication. We found that peropsin in mollusks is almost always single copy—the only exception here is the 2 copies in the blood clam, *S. kagoshimensis*—but prone to loss (Fig. 3). Peropsin has been lost at least 7 times in mollusks, including in squid and nautilus, a gastropod clade containing Caenogastropoda and Heterobranchia, and numerously across bivalves: Pectinida, Mytiloida, Unionida, Cardiida (as evidenced by *Gari tellinella*), and some venerids (Fig. 3). The chiton genome also lacks peropsin, which suggests gene loss but a more robust phylogeny of the RGR/retinochrome/peropsin opsin clade would be needed for clarity and help reconstructing the history of functional divergence between these different photoisomerase opsins.

#### Repeated Xenopsin Expansions Contribute to Large Opsin Repertoires in Mollusks

We estimated 175 xenopsin duplication events, 3 to nearly 90 times higher than any other opsin type (Table 3). Xenopsins appear to be particularly prone to expansion in bivalves. For example, in Pteriomorphia, the ark clams (Arcida), scallops (Pectinida), mussels (Mytilida), and oysters (Ostreida) all experienced distinct, multiple rounds of xenopsin duplication (Fig. 4a). In another bivalve lineage, xenopsins account for most of the opsin sequences we recovered from the 2 *Dreissena* genomes (44 out of 54; 42 out of 63). The vestigastropods also have multiple rounds of xenopsin duplication, not shared by other gastropods.

This little-known opsin clade has only recently been recognized as a distinct opsin type, in part, because they are absent in chordates and arthropods. Xenopsin expression has been observed in larval eyes of the chiton *Leptochiton asellus* (Vöcking et al. 2017), flatworm (Rawlinson et al. 2019), and the bryozoan (Döring et al. 2020). Xenopsin is also expressed in adult eye rhabdomeric photoreceptors of the land slug, *Limax valentianus* (Nishiyama et al. 2019). In all four of these studies, xenopsin was co-expressed with r-opsins, and notably c-opsin in the case of the flatworm (Rawlinson et al. 2019), demonstrating that xenopsin and c-opsins are not mutually exclusive groups of opsins, as had initially been hypothesized (Vöcking et al. 2017). Rawlinson et al. (2019) also showed, in vitro, that xenopsins couple with G_αi_ to drive phototransduction, which was supported by (Sakai et al. 2022) who also demonstrated blue-light sensitivity and bistability in xenopsin from a chiton, brachiopod, platyhelminth, and chaetognath. These results point toward a scenario where xenopsin photopigments play an important role in visual processing in protostomes, including mollusks, but the exact nature of their function remains unclear, particularly for eyeless species.

#### Go-Opsin Numbers Differ Greatly Across Gastropods

The G_o_-opsins were also prone to expansions in gastropods and mytiloid bivalves, though to a lesser extent than xenopsins (Fig. 3, Table 3). This type of opsin was first discovered in scallop eyes and was notably expressed in the ciliary cells of the distal retina (Kojima et al. 1997). These opsins differ in their coupled G-protein, but also in that G_o_-opsin-based photoreception leads to cell membrane hyperpolarization, rather than depolarization found in r-opsin systems (Table 1). Other than their effect on cell membrane potential and G-protein, very little is known about G_o_-opsins, but they have been tied to digestion regulation via control of pyloric opening in sea urchin larvae (Yaguchi and Yaguchi 2021), shadow response in annelid (Ayers et al. 2018), and detection of moving objects in scallops (Speiser et al. 2016).

The G_o_-opsins were typically present in high numbers in various gastropod lineages. The species with the highest number of G_o_-opsins (8–10 sequences) were the Vetigastropoda, with true limpets and abalone sharing numerous rounds of duplication (Fig. 3). In addition to xenopsins in some bivalves, the G_o_-opsins in abalone represent some of the most abundant opsins in mollusks. Our results also point to strong clade-level discrepancies between G_o_-opsins abundance within the gastropods (Fig. 3). We found 3 to 7 G_o_-opsins in caenogastropods, while Heterobranchia had a reduced set with both Neogastropoda and Planorbidae having a single G_o_-opsin per species and a loss of G_o_-opsin entirely in the Stylommatophora land snails. Species in this clade of land snails have the smallest opsin repertoire that we observed, outside of the cephalopods. The reduced opsin diversity in land snails could be related to transitions to terrestrial habitats that have different light availability relative to aquatic relatives. Terrestrialization in gastropods has occurred upwards of 30 times (Vermeij and Watson-Zink 2022), yet genomic data has thus far only been collected from this single transition. A much greater genomic sampling of gastropods will be needed to formally test whether transitions to land are related to opsin repertoire in gastropods.

#### Rhabdomeric Opsin Duplication is Rare in Mollusks But Not Restricted to Scallops

The opsins most consistently recovered in the genomes from this phylum-wide search were the 2 clades of rhabdomeric or r-opsins: the “canonical” (G_q_-coupled, invertebrate visual opsin) and “noncanonical” (Fig. 1). The “noncanonical” clade was only recently described in Ramirez et al. (2016) as a distinct group from the r-opsins that include arthropod and molluscan visual opsins and chordate melanopsin (collectively referred to as “canonical” in Ramirez et al. 2016). The noncanonical r-opsins have been found in mollusks and other lophotrochozoans, hemichordates, and nonvertebrate chordates, indicating that this r-opsin split predates metazoan—or at least bilaterian—diversification, lending support for nomenclatural distinction. Whether the noncanonical r-opsins have similar functional properties as the better described canonical r-opsins is unknown. Other than the deep-sea lineages that completely lacked opsins, only 1 molluscan species appears to not have a noncanonical r-opsin, the pearl oyster *Pinctada imbricata*, though we found a noncanonical r-opsin in the congener *Pinctada fucata*. Most species in our dataset had 1 to 3 noncanonical r-opsins, but we observed 12 copies in *Dreissena* and a similarly large expansion in some venerid bivalves (Fig. 3). Most of the noncanonical r-opsins have the HPK tripeptide motif located in the fourth extracellular loop (Fig. 2a), which is a hallmark G_αq_-coupled opsins (Plachetzki and Oakley 2007), so it is likely that these opsins function similarly to the canonical r-opsins (see Table 1 for details).

Canonical r-opsin was recovered from every genome—excluding the deep-sea lineages with total opsin loss—except for the sea slug *Aplyisa californica*, in which we found evidence of a pseudogenized remnant sequence. Given the established role of canonical r-opsins in visual processing for invertebrate bilaterians, the evolution of these opsins may be particularly tied to eye complexity and photic environment. Duplication and specialization of canonical r-opsins in arthropods are very common (Cronin and Porter 2014). In jumping spiders, for example, different r-opsin genes are expressed in the principal eye and 3 secondary eyes (Nagata et al. 2012). In the most extreme example yet, mantis shrimp have been shown to express 33 r-opsins in their compound eyes, with several distinct combinations of opsins sensitive to ultraviolet, long, short, and medium wavelengths of light observed across numerous distinct photoreceptor types (Porter et al. 2020). This degree of r-opsin diversification is not seen in mollusks. In fact, the majority (58/80) of mollusk species examined here have a single canonical r-opsin sequence. However, it is worth noting that arthropods lack xenopsins and G_o_-opsins (Ramirez et al. 2016), the 2 most expansion-prone groups of opsins in mollusks according to our findings.

Despite the overall rarity of canonical r-opsin duplication in mollusks, an expansion in scallops represents a compelling case for opsin recruitment in the evolution of novel eyes. A single r-opsin duplication was initially identified in the bay scallop, *Argopecten irradian*s, a notable exception for mollusks at the time (Serb et al. 2013). With transcriptomic sequencing, four r-opsins were eventually identified in *A. irradians*, which differed in expression levels and had some notable sequence differences at critical amino acid sites (Porath-Krause et al. 2016). Analyses of additional scallop genome assemblies have also identified four r-opsins, which tend to have eye-biased gene expression levels (Li et al. 2017; Wang et al. 2017).

We recovered four r-opsins in all 5 species of scallops surveyed here (Fig. 2b, Fig. 3). The 3 intronless r-opsins in scallops form their own clade, demonstrating that the first r-opsin duplication in scallops (apparently a retrotransposition event) likely occurred in the ancestor for all scallops (Porath-Krause et al. 2016). We also observed a second series of r-opsin duplication events exclusive in sea mussels (Fig. 2b, Fig. 3). The four species of *Mytilus* along with *Gigantidas platifrons*, *Modiolus philippinarum*, and *Perna viridis* all have 3 r-opsins and none of these species have eyes as adults, bringing the hypothesis of r-opsin recruitment for visual processes into question. A notable difference between the scallop and mussel r-opsin expansions is the possible functional divergence of the scallop duplicates, as indicated by their E/DRY motif, a sequence in the third TM important for stabilizing GPCR inactive-state confirmation (Rovati et al. 2007). In the scallop r-opsin clades, we see the following motifs: DRY, DRC, DRF, while all the mussel r-opsins have DRY (Fig. 2b). In fact, DRY was found in all other r-opsins recovered from mollusk genomes in this study (see logo in Fig. 2a), except for the 2 *Pinctada* pearl oyster species (Fig. 2b).

### Mollusks Have Up To 6 Types of CRY-PL Proteins

**Fig. 5.**
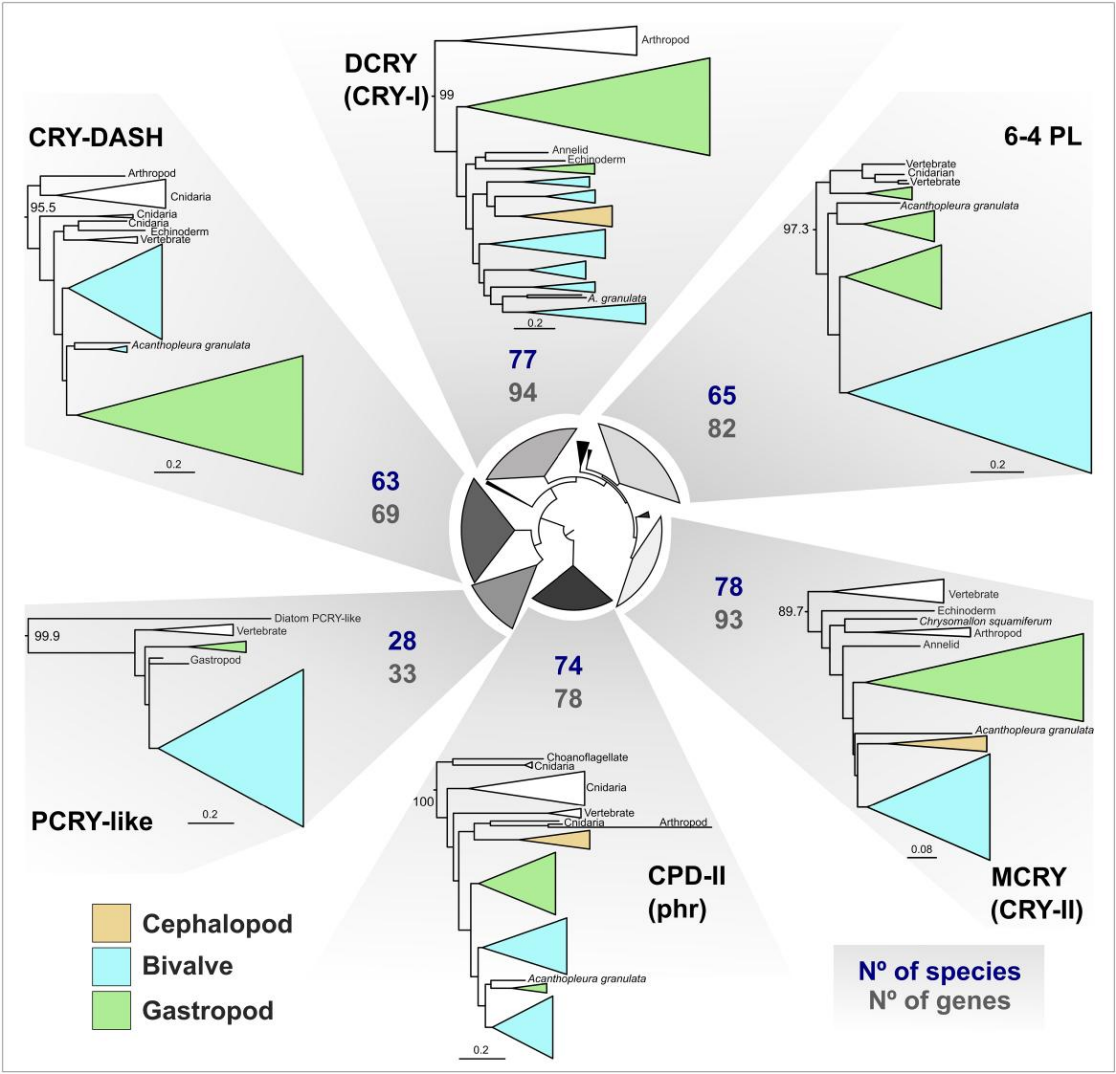
CRY-PL clades are similar in total size and number of species represented. Subtrees of mollusk containing CRY-PL clades displayed from the IQ-TREE2 ML CRY-PL phylogeny (LG + R8 amino acid substitution model). For each of 6 CRY-PL types found in mollusks, the total number of sequences in the tree (N^o^ of genes) and species (N^o^ species) with that gene are displayed. Clades are collapsed according to taxonomic clades (e.g. bivalves) and color coded by mollusk class. CRY-PL names in parentheses (e.g. “CRY-I”) reflect common nomenclature for animal proteins used in text. UF-bootstrap support values shown at base of named of CRY-PL clades. See supplementary fig. S12b, Supplementary Material online for branch support values for circular inner tree and mollusk-specific clades in supplementary fig. S11, Supplementary Material online.

Using curated classified reference protein sets and the BITACORA protein prediction pipeline, we identified 440 CRY-PL gene models from 80 molluscan genomes (Fig. 5). Our phylogenetic analysis of the mollusk sequences from CRY-PL placed all 440 sequences (excluding 4 likely contaminant sequences) into the following 6 classifications: CPD-II (phr), CRY-DASH, PCRY-like, DCRY (CRY-I), 6-4 PL, and MCRY (CRY-II) clades. Broadly, our CRY-PL ML phylogeny includes 10 major clades with high statistical support that also included sponge CRYs, AnthoCRYs, AnthoCRY-II, and CRY4 (Fig. 5). We did not resolve the PCRY/PCRY-like/CPD-III/CPD-I relationships (hence the collapsed clade in Fig. 5), as the phylogeny was constructed to classify the predicted sequences from BITACORA output and not meant to be an exhaustive reconstruction of the entire CRY-PL family. However, the PCRY-like clade does include a monophyletic mollusk group (supplementary fig. S11, Supplementary Material online), indicating strong support for our classification of those sequences. Broadly, the CRY-PL topology here (Fig. 5) recapitulates what has been reported from other studies (e.g. Oliveri et al. 2014; Deppisch et al. 2022; Vicedomini et al. 2022).

Most mollusk species have CRY-I, CRY-II, CRY-DASH, CPD-II (i.e. phr), and 6-4 PL (Fig. 3). We found that phr and the animal cryptochromes (CRY-I, CRY-II), were present across all molluscan classes and in nearly all species, while CRY-DASH and (6-4)PLs are absent in cephalopods and some terrestrial gastropods, with occasional losses from species in well-represented taxonomic classes (e.g. CRY-DASH absence in Dreissenidae, Bivalvia) (Fig. 3). The PCRY-like group is restricted to specific molluscan lineages, including 2 gastropod clades: Trochidae “top-snails” and Conidae “cone-snails,” along with most bivalves, though it appears to have been lost in Pectinidae while maintained in other pteriomorphian families (PCRY-L in Fig. 3). This somewhat spotty recovery of PCRY-like in mollusks is similar the findings in Deppisch et al. (2022), who suggest that the presence of PCRY-like in the oyster *Crassostrea gigas* and other marine mollusks may be from horizontal gene transfer (HGT), as bacterial genes have been identified in marine metazoans, like the jellyfish *Nematostella vectensis* (Starcevic et al. 2008). However, our phylogeny recovers a monophyletic molluscan PCRY-like. Furthermore, the PCRY-like clade in Deppisch et al. (2022), too, has a monophyletic group of metazoan PCRY-like sequences, with a vertebrate clade and mollusk—annelid—arthropod clade sister to it. These results are not indicative of HGT unless it occurred in the last common ancestor of metazoan. Instead, the patterns of presence and absence of this sequence across mollusks and other metazoans likely reflect frequent loss, as proposed by Oliveri et al. (2014) in the first phylogenetic description of the widespread occurrence of PCRY-like sequences across bilaterians. Still, a greater survey of microbial genomes for PCRY-like sequences could help clarify the origin of this gene outside of plants. Other than evidence of rhythmic expression of this gene from zebrafish (Oliveri et al. 2014), virtually nothing else is known about the function of this protein.

#### Cryptochrome Abundance is Highly Restricted Relative to Opsins

We observed very few instances of retained cryptochrome duplication in the molluscan genomes we surveyed (Fig. 3). Second copies of each cryptochrome were identified spuriously across our species tree, but we did not observe any apparent shared duplication within taxonomic clades as we did with opsins. The only species that we recovered more than 2 copies for a given cryptochrome was in the zebra mussel, *Dreissena polymorpha*, in which we also found the highest number of opsins. In *D. polymorpha*, we found evidence of 3 (6-4)PLs and 5 CRY-II. In the congener species *D. rostriformis*, the quagga mussel, did not have the same CRY-II duplications, but the 2 species share a (6-4)PLs duplication. The annotations for the quagga and zebra mussel genome assemblies both reported exceptionally high numbers of predicted protein sequences for metazoans and numerous gene family expansions were described in the zebra mussel, though the authors acknowledged that their results are likely an overestimation (Calcino et al. 2019; McCartney et al. 2022).

Though cryptochromes do not diversify in a similar manner to opsins, there were a few notable parallels between opsin and cryptochrome loss in our results. Cephalopoda lost neuropsin and G_o_-opsins and appears to have lost CRY-DASH and (6-4)PLs, 2 UV-induced DNA-damage repair PL in the CRY-PL. *Nautilus* also lacks phr while it is present in the other cephalopod genomes. Similarly, the group of land snails with reduced opsin repertoires, including loss of G_o_-opsin, are also mostly missing CRY-DASH and (6-4)PLs. The exceptions being *Cepea nemoralis*, which has both genes, and *Arion vulgaris*, which has a DRY-DASH. The dreissenid clams also lost both CRY-DASH and G_o_-opsins. Therefore, CRY-DASH and G_o_-opsin have been jointly lost at least once in bivalves, cephalopods, and gastropods. Determining if a biological connection exists between CRY-DASH and G_o_-opsins in mollusks will be important to our understanding of photopigment functions. Cryptochrome loss was also evident in the same deep-sea lineages lacking opsins (see below). The 2 deep-sea snails only had CRY-II, which, based on the mammalian circadian clock, functions independent of light (Griffin et al. 1999), and the deep-sea clam *Archivesica marissinica* lacked every CRY-PL sequence we searched for.

#### Opsin and Cryptochrome Loss in Deep-Sea Mollusks

The most apparent connection between opsin content and molluscan biology and ecology we found was complete opsin loss in 3 deep-sea mollusks. Two gastropods, the scaly foot snail, *Chrysomallon squamiferum* and *Gigantopelta aegis,* and a venerid clam, *Archivesica marissinica*, appear to have no opsin sequences in their genomes. None of the 3 opsinless species had particularly low numbers of BITACORA-predicted genes with opsins as input, indicating that this is not a failure of our approach. For *A. marissinica*, 177 genes were predicted (vs. 243 ± 108 in bivalves), *C. squamiferum* and *G. aegis* had 162 and 186, respectively (vs. 224 ± 117 in gastropods), indicating that the quality of the genomes and annotation approach were not biased. Furthermore, we detected many opsins in close relatives of these species, indicating that the phylogenetic distance to the reference opsin set was not preventing us from predicting opsins in these genomes. Additionally, we used tblastn to specifically query opsin amino acid sequences against these genomes and were unable to identify evidence of remnant opsin sequences (e.g. pseudogenes). Similarly, these 2 deep-sea snails have lost all members of the CRY/PL family except for the light-insensitive CRY-II, and the bivalve, *A. marissinica*, lacks any sequence from this protein family. Cryptochrome loss has also been observed in at least 1 deep-sea fish species, the coelacanth (Deppisch et al. 2022).

The 2 snail species belong to a single deep-sea transition in Peltospiridae, so our data only capture opsin loss in 2 distinct deep-sea mollusk lineages. Opsin loss has also been observed in deep-sea anthozoan cnidarians (Gornik et al. 2020). Loss of opsins in deep-sea mollusks and cnidarians is a stark contrast to some deep-sea teleost fish, where independent rhodopsin expansions—including up to 38 copies in the silver spinyfin—have been observed (Musilova et al. 2019). In the water beetle family Dytiscidae, independent transitions to subterranean life are associated with loss/decay of opsin and other phototransduction genes (Langille et al. 2022). Testing whether other proteins involved in phototransduction are missing or possibly nonfunctional from these opsinless deep-sea molluscan lineages will be a useful approach to exploring the genomics of trait decay.

From our taxonomic sampling, we cannot determine what factors lead to opsin loss in some deep-sea lineages and not others. Surprisingly, 2 other deep-sea mollusks in our dataset, the gastropod *Phymorhynchus buccinoides* and bivalve *Gigantidas platifrons* have similar numbers of opsin and CRY-PL genes when compared to their closest relatives. *Phymorhynchus buccinoides* occur in cold seeps versus the hydrothermal vents that support the scaly-foot snail. Both habitats should favor chemosensation over vision, and indeed, *C. squamiferum* has lost its eyes and numerous *Phymorhynchus* species, too, lack eyes (Zhang and Zhang 2017). In the deep-sea mussel *Bathymodiolus azoricus*, a close relative of *G. platifrons*, cryptochromes and other genes involved in circadian rhythm is expressed in a rhythmic fashion in the natural hydrothermal vent environment, apparently driven by tidal signal (Mat et al. 2020). Depth at which a species is found could be a parameter for opsin retention vs. loss. The scaly-foot snail is found at depths near 3,000 m while *P. buccinoides* occurs at 1,160–1,190 m. However, the deep-sea clam, *A. marissinica* is reported from 1,400 m and has completely lost its opsins. Another possible factor for loss of opsin in some species but not others could be the time since transitioning the aphotic deep-sea environments, as has been seen with repeated colonization of caves by teleost fish (Niemiller et al. 2013). Testing whether these species that lack opsins or cryptochromes can still perceive light will be critical to better understanding the degeneration of visual and light-sensing systems in deep-sea animals.

### What Accounts for Differing Opsin Content in Mollusks?

From our genomic survey of photopigment genes in mollusk genomes, no apparent connection to eye or eye specialization emerges (Fig. 3), except for the loss of opsins and cryptochromes in some deep-sea lineages. We observed the highest numbers of opsins in eyeless bivalve species and the fewest number of opsins in cephalopods, a lineage with sophisticated eyes. Therefore, the degree of specialization in light-sensing organs appears to be independent of the diversity and abundance of opsins in mollusks, as was reported recently by De Vivo et al. (2023). The question is then, what other organismal and environmental factors drive opsin evolution for these species?

In addition to the increasing number of recorded extraocular functions for opsins, a growing body of literature points to numerous light-independent functions for opsins, suggesting that how we view opsins should change to describe them as a broad sensory-driven signaling molecule (Feuda et al. 2022). It has been argued that light-absorption may not even be the “original” function of opsins (Leung et al. 2020; Pisani et al. 2020; Feuda et al. 2022). Because opsins may be used to regulate a variety of physiological processes, taking in more than just light, we need to consider the possibility that the extensive diversification of opsins in species such as venerid clams and *Dreissina* species reflects selection on broad multisensory receptors.

Light, chemical, and other environmental cues may be especially important for animals with distinct life stages. The reduced sets of opsins in bryozoans and platyhelminths relative to mollusks and other lophotrochozoans may be connected to such life-stage distinction (De Vivo et al. 2023). The possibility that life cycle complexity influences opsin family evolution could extend to within-Mollusca differences in opsin diversity. Unlike most cephalopods and gastropods in freshwater or terrestrial environments that have direct development, most marine bivalves and gastropods have several morphologically distinct larval stages. These mobile larval stages are the main mechanism for dispersal for many marine mollusks, which after a “veliger” stage metamorphose and settle into a sessile adulthood. As a result, it is important for these species to be “choosy” when identifying appropriate substrates. Previous work has shown that bivalve species, like *Mytilus*, are highly selective of where to settle (e.g. Carl et al. 2012). Interestingly, chemical cues that likely induce abalone settlement and metamorphosis involve unidentified TM G-protein coupled receptors, the same superfamily of opsin (Baxter and Morse 1987, 1992). Our study uncovered large opsin repertoires in marine species, with abalone having the highest numbers among gastropods. We think that opsins, as TM receptors, are worthwhile proteins to explore in the context of life-stage triggers and “decisions” on settling in mollusks.

Among the most immediate next steps to better understand opsin use in mollusks is determining where and when these genes are expressed, e.g. different tissues and developmental stages. This question can be addressed with tissue-specific and single-cell RNA-seq, along with in situ RNA hybridization and immunohistochemistry. Whether these opsin sequences encode potential photopigments can be determined with heterologous expression assays in cell culture (e.g. Faggionato and Serb 2017; Smedley et al. 2022). There is the possibility that (some) of these molluscan opsins have the potential to detect light but serve other functions, i.e. multimodality, and can first be assessed from protein-ligand predictions based on protein models, such as the AlphaFill algorithm applied to Alphafold models (Hekkelman et al. 2023). Given the successful application of CRISPR gene editing in bivalves (Yu et al. 2019; Jin et al. 2021), cephalopods (Crawford et al. 2020; Ahuja et al. 2023), and gastropods (Perry and Henry 2015; Abe and Kuroda 2019), there is also potential to incorporate gene knock-out experiments into future explorations of molluscan opsin functions. Using molecular evolutionary analyses of opsin sequence to detect positively selected sites associated with photic and other environmental variables (e.g. Castiglione et al. 2017; Sondhi et al. 2021; Van Nynatten et al. 2021) will also be a powerful approach to characterizing molluscan opsins as more genomic data becomes available.

## Conclusion

Our results reveal that mollusks vary greatly in the types and abundance of proteins involved in light-sense in their genomes. Our phylogenetic analysis of predicted opsin sequences from 80 molluscan genome assemblies supported at least 7 major opsin clades with species total opsin counts ranging from zero to 63 copies. We find that some types of opsins, like retinochrome, are evolutionarily static, characterized by rare cases of duplication, while other opsins repeatedly expand in lineage-specific manner, such as xenopsin. These results stand in stark contrast to another group of photopigments, cryptochromes, which have not diversified within mollusks. We found that some deep-sea species lack opsins and cryptochromes, indicating these proteins can be dispensable for mollusks in aphotic environments. Other than deep-sea-related loss of opsins, we see no clear connection between opsin diversity and photic environments but discuss possible connections to terrestriality. The bivalves in this study, most of which lack eyes, tended to have the greatest number of opsins, with repeated lineage-level expansions accounting for the observed diversity in opsin repertoire. The abundance of opsins in these eyeless species raises important questions about what function these genes play in how mollusks sense their environment.

## Supplementary Material

msad263_Supplementary_Data

## Data Availability

All gene predictions from BITACORA, sequences used in opsin and CRY-PL phylogenetic analyses, alignments, and phylogenetic trees are available through FigShare (https://doi.org/10.6084/m9.figshare.23593641).
