# Supplementary material for: Molluscan Genomes Reveal Extensive Differences in Photopigment Evolution Across the Phylum
Source: Mol Biol Evol. 2023 Dec 1;40(12):msad263. doi: 10.1093/molbev/msad263 (PMC10733189; doi:10.1093/molbev/msad263)
Supplement: msad263_Supplementary_Data [file msad263_supplementary_data.zip › McElroy_et_al_MBE_supplemental-figures.pdf]

SUPPLEMENTAL FIGURES

Figure S1. Histogram and density plots of BUSCO % complete scores of mollusk genomes based on the metazoan_obd10 and mollusca_obd10 databases. Vertical dashed lines represent mean values for each set of results.

Figure S2. Classification of molluscan opsins used as reference sequences in BITACORA. Maximum likelihood phylogeny of 109 reference molluscan opsin sequences (blue text) 86 molluscan opsin sequences from Ramirez et al. (2016). Phylogeny generated with IQtree2 (LG+F+R10). Sequences used for BITACORA reference database include species code and original source name (Obim: *Octopus bimaculoides*, Acal: *Aplysia californica*, Bgla: *Biomphalaria glabrata*, Lgig: *Lottia gigantea*, Pcan: *Pomacea canaliculate*, Cgig: *Crassostrea gigas*, Pmax: *Pecten maximus*, Sbro: *Scapharca broughtonii*, Scon: *Sinonovacula constricta*). "Scon-opnGxS#" are xenopsins exclusive to Scon. See Table S4 for classification names of reference opsins from this tree. Tree rooted with outgroups from Vocking et al. (2017).

Figure S3. Maximum likelihood species tree generated with IQtree2 based on a partitioned amino acid supermatrix from 899 complete single copy BUSCO sequences (metazoan_obd10) recovered from at least 60/80 species (285,575 total sites). Branch support values are SH-aLRT % support (with 1000 replicates)/aBayes probability/UFBoot support % (with 1000 replicates). Branches without support shown are 100/1/100 support.

Figure S4. Classification of molluscan CRY-PL used as reference sequences in BITACORA. Maximum likelihood of 43 molluscan CRY-PL sequences with 11 landmark CRY-PL sequences (bold text) from Deppisch et al. 2022. Phylogeny generated with IQtree2 (LG+R5). Branch support values are SH-aLRT % support (with 1000 replicates)/aBayes probability/UFBoot support % (with 1000 replicates).

Figure S5. Maximum likelihood phylogeny of molluscan opsin amino acid sequences generated with IQtree2 (LG+F+R10). Branch values are SH-aLRT % support (with 1000 replicates)/aBayes probability/UFBoot support % (with 1000 replicates). Tree rooted with outgroups from Vocking et al. (2017). Cartoons summaries of subfamilies are representative of the number of sequences (number of sequences in each subfamily noted). The "unclassified tetraopsin" clade contains the "unclassified" sequence from *Lottia gigantea* in the reference opsin dataset and is exclusive to the chiton, *Acanthopleura granulata* and other gastropods. All but 2 sequences – each from chiton – are classifiable according to their inclusion in highly supported clades. *clade S of the xenopsins contains only the "Scon-opnGxS#" xenopsins along with sequences from the Heteroconchia bivalves.

Figure S6. Maximum likelihood phylogeny of opsin amino acid sequences generated with IQtree2 (LG+F+R10). BITACORA opsins from Figure S5 along with opsins from additional taxa outlined in Table S6. Branch values are SH-aLRT % support (with 1000 replicates)/aBayes probability/UFBoot support % (with 1000 replicates). Mollusk opsin clades highlighted with light blue and the origin of the r-opsins and tetraopsins are highlighted because of the numerous subclades present. Note: same tree as used in Figure S1.

Figure S7. Opsin phylogeny from Figure 1 and S6 with all branches not collapsed. Grey shading indicates non-molluscan opsin clades, colored shading is the same as Figure S5 with adjusted xenopsin color for clade a vs. b.

Figure S8. Maximum likelihood phylogeny of opsin amino acid sequences generated with IQtree2 (LG+F+R10). BITACORA opsins from Figure S5 along with opsins from additional taxa outlined in Table S6. Branch values are SH-aLRT % support (with 1000 replicates)/aBayes probability/UFBoot support % (with 1000 replicates). Mollusk opsin clades highlighted with light blue and chiton sequences (Agra-opsin-X) highlighted in dark blue. Mollusk opsin clades highlighted with light blue and the origin of the r-opsins and tetraopsins are highlighted because of the numerous subclades present. Bathyopsin subtree included to compare with Figure S8 for chiton sequence classification.

Figure S9. Classification of unknown chiton opsin sequences. Maximum likelihood phylogeny of a broad sample of opsin subfamilies, including several non-molluscan clades to classify two opsin sequences more precisely from the chiton, *Acanthopleura granulata*. Two opsins from *A. granulata* did not group with any other molluscan opsin subfamily (Figure 1, Agra_opsin-086.t1 and Agra_opsin-128.t1). Anthozoan-I, anthozoa-II, bathyopsin (Ramirez et al. 2017), c-opisn, Chaopsin (Ramirez et al. 2017), cnidopsin, and ctenopsin sequences included with the molluscan reference opsins (figure way to reference) and *A. granualata* opsins for maximum likelihood analysis with IQtree2 (substitution model). Mollusk opsin clades highlighted with light blue and chiton sequences (Agra-opsin-X) highlighted in dark blue. Brach support from -B 1000 --alrt 1000 –abayes.

Figure S10. GeneRax reconciled retinochrome tree. Molluscan classes highlighted with same color gradients as Figure S3 and Bivalvia and Cephalopoda clades named to help distinguish groups with retinochrome duplication. Reconciled tree visualization generated with ThirdKind to show locations of gene duplication and loss given at nodes and tips of species tree. Blue line traces evolutionary history for each gene.

Figure S11. Maximum-likelihood phylogeny of CRY-PL from Figure 5 with no collapsed branches. Tree generated with IQ-TREE2 (LG + R8). Branch values are SH-aLRT % support (with 1000 replicates)/aBayes probability/UFBoot support % (with 1000 replicates). Clades containing mollusk sequences shaded in blue, with any non-molluscan sequences mixed in identified with red text. Support values underlying classification of mollusk CRY-PLs are bolded. CRY-PL names in parentheses (e.g., “CRY-I”) reflect common nomenclature for animal proteins, used in text.

Figure S12. Support values for circular trees from **a)** Figure 1 and **b)** Figure 5. Phylogenetic relationships of opsin and cryptochrome/photolyase (CRY-PL) protein families. Mollusks have **a)** 7 types of opsins and **b)** 6 types of CRY-PL proteins. Both trees generated by maximum likelihood with IQtree2 based on amino acid sequence alignments using **a)** LG+F+R10 and **b)** LG+R8 substitution models selected based on Bayesian information criterion scores. Branch values are SH-aLRT % support (1000 replicates)/aBayes probability/UFBoot support % (1000 replicates). **a)** rooted with non-opsin G-protein coupled receptor outgroups from Vocking et al. (2017). Mollusk-containing clades shaded in blue and bold text further specificity groups with mollusk sequences in cases of multiple small clades grouped together. “Unclassified tetraopsin” in **a)** is a mollusk-specific clade containing sequences from the chiton, *Acanthopleura granulata* and Psilogastropoda (Cunha and Giribet 2019) gastropods that groups with neuropsin and Go-opsin but its placement is less certain. CRY-PL names in parentheses (e.g., “CRY-I”) reflect common nomenclature for animal proteins, used in text.

Figure S1

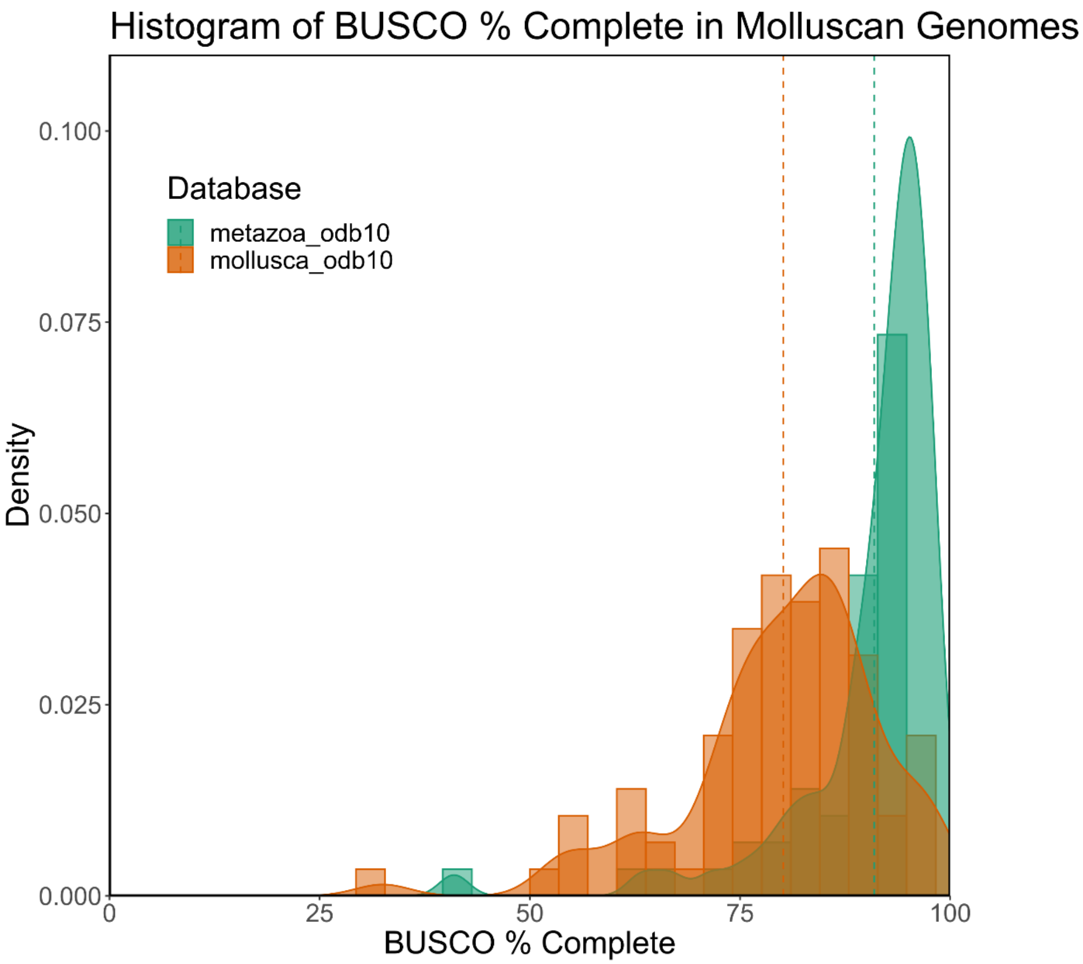

Histogram of BUSCO % Complete in Molluscan Genomes
Database
metazoa_odb10
mollusca_odb10
Density
0.100
0.075
0.050
0.025
0.000
0
25
50
75
100
BUSCO % Complete

Figure S2

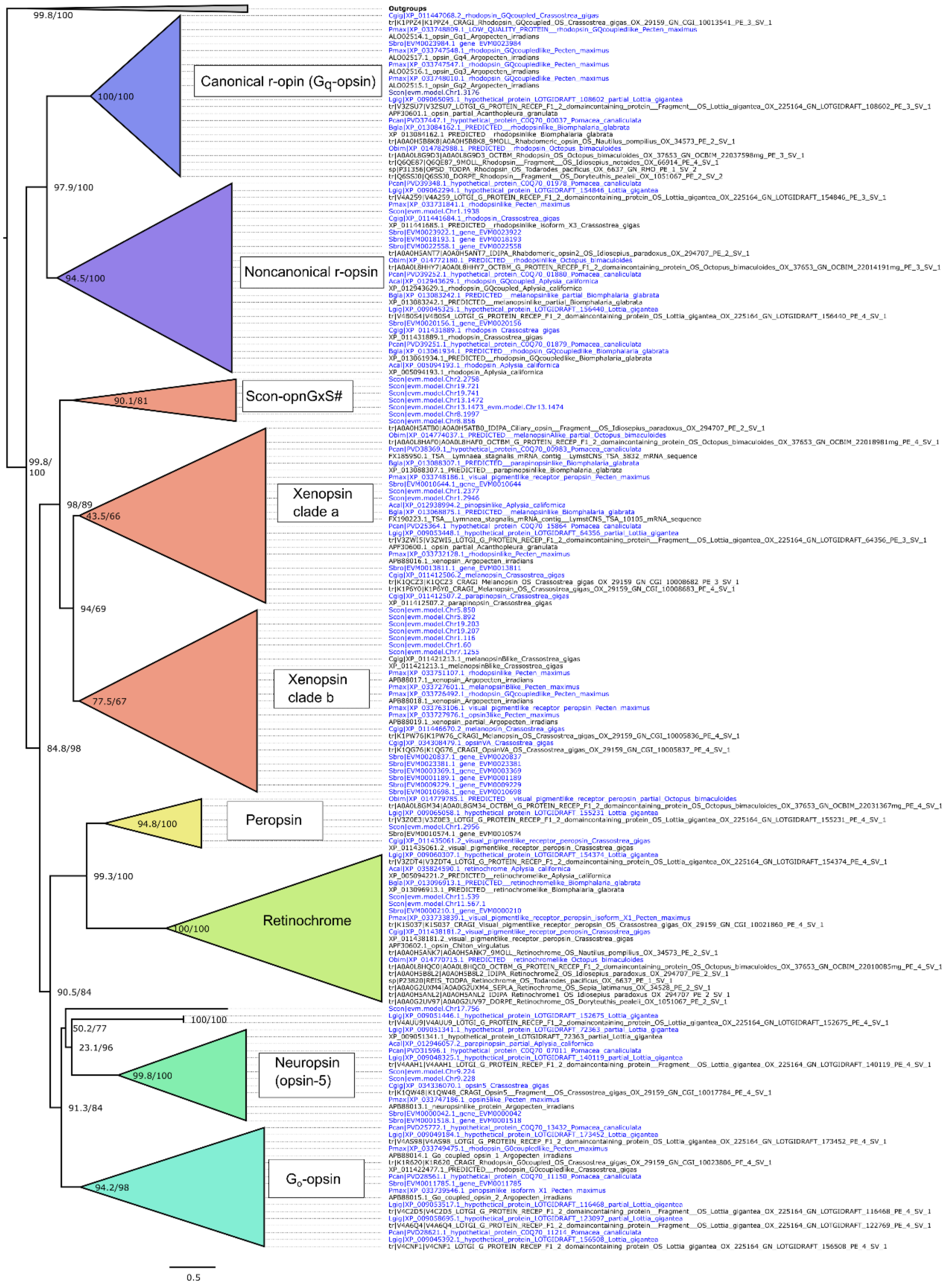

Outgroups
99.8/100
Canonical r-opin (Gq-opsin)
100/100
97.9/100
Noncanonical r-opsin
94.5/100
Scon-opnGxS#
90.1/81
99.8/
100
98/89
43.5/66
Xenopsin
clade a
94/69
Xenopsin
clade b
77.5/67
84.8/98
Peropsin
94.8/100
99.3/100
Retinochrome
100/100
90.5/84
100/100
50.2/77
23.1/96
Neuropsin
(opsin-5)
99.8/100
91.3/84
Go-opsin
94.2/98
0.5

Figure S3

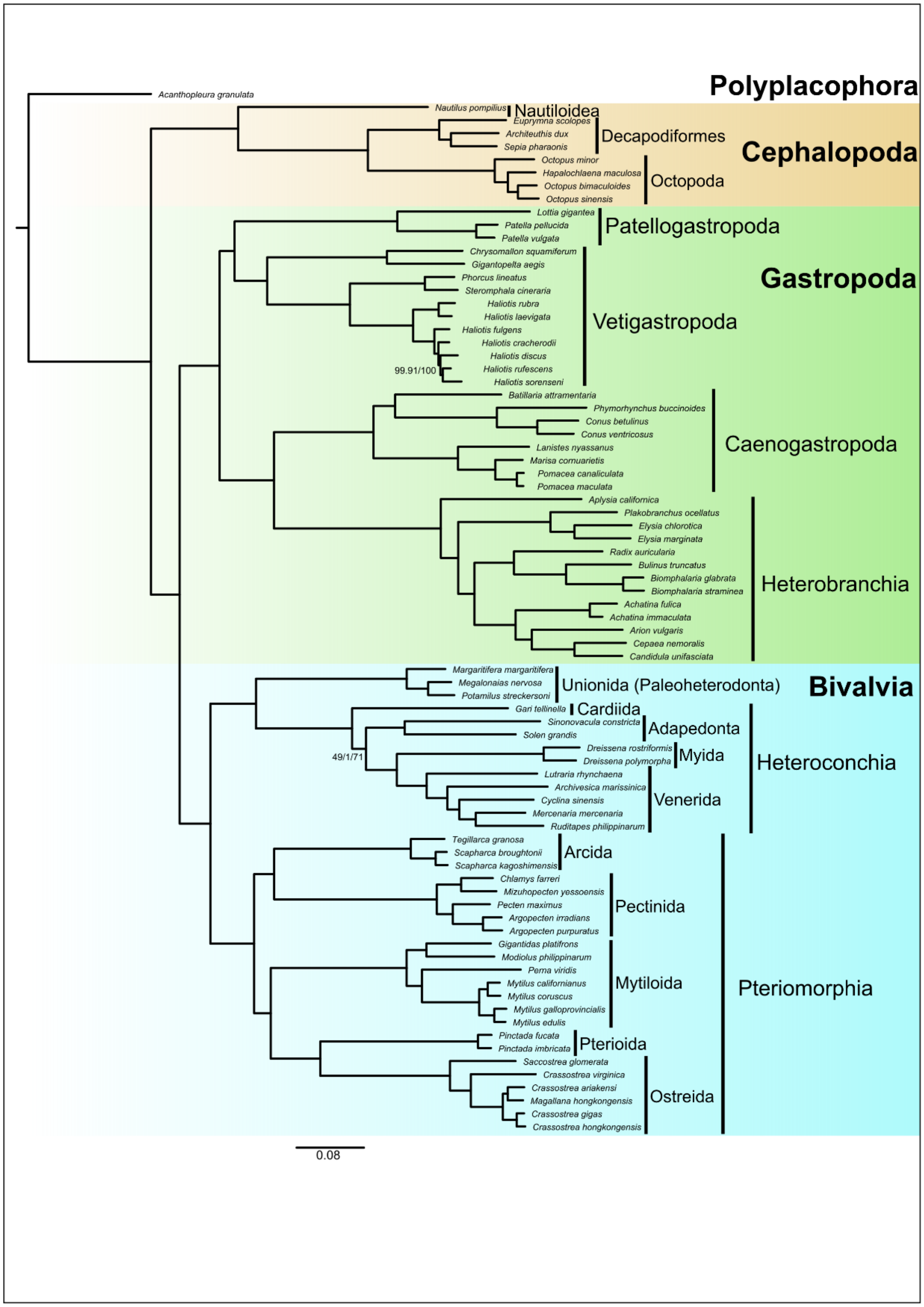
Polyplacophora
Acanthopleura granulata
Cephalopoda
Nautiloidea
Nautilus pompilius
Decapodiformes
Euprymna scolopes
Architeuthis dux
Sepia pharaonis
Octopoda
Octopus minor
Hapalochlaena maculosa
Octopus bimaculoides
Octopus sinensis
Gastropoda
Patellogastropoda
Lottia gigantea
Patella pellucida
Patella vulgata
Vetigastropoda
Chrysomallon squamiferum
Gigantopelta aegis
Phorcus lineatus
Steromphala cineraria
Haliotis rubra
Haliotis laevigata
Haliotis fulgens
Haliotis cracherodii
Haliotis discus
Haliotis rufescens
Haliotis sorenseni
99.91/100
Caenogastropoda
Batillaria attramentaria
Phymorhynchus buccinoides
Conus betulinus
Conus ventricosus
Lanistes nyassanus
Marisa cornuarietis
Pomacea canaliculata
Pomacea maculata
Heterobranchia
Aplysia californica
Plakobranchus ocellatus
Elysia chlorotica
Elysia marginata
Radix auricularia
Bulinus truncatus
Biomphalaria glabrata
Biomphalaria straminea
Achatina fulica
Achatina immaculata
Arion vulgaris
Cepaea nemoralis
Candidula unifasciata
Bivalvia
Unionida (Paleoheterodonta)
Margaritifera margaritifera
Megalonaias nervosa
Potamilus streckersoni
Heteroconchia
Cardiida
Gari tellinella
Adapedonta
Sinonovacula constricta
Solen grandis
Myida
Dreissena rostriformis
Dreissena polymorpha
49/1/71
Venerida
Lutraria rhynchaena
Archivesica marissinica
Cyclina sinensis
Mercenaria mercenaria
Ruditapes philippinarum
Pteriomorphia
Arcida
Tegillarca granosa
Scapharca broughtonii
Scapharca kagoshimensis
Pectinida
Chlamys farreri
Mizuhopecten yessoensis
Pecten maximus
Argopecten irradians
Argopecten purpuratus
Mytiloida
Gigantidas platifrons
Modiolus philippinarum
Perna viridis
Mytilus californianus
Mytilus coruscus
Mytilus galloprovincialis
Mytilus edulis
Pterioida
Pinctada fucata
Pinctada imbricata
Ostreida
Saccostrea glomerata
Crassostrea virginica
Crassostrea ariakensis
Magallana hongkongensis
Crassostrea gigas
Crassostrea hongkongensis
0.08

Figure S4

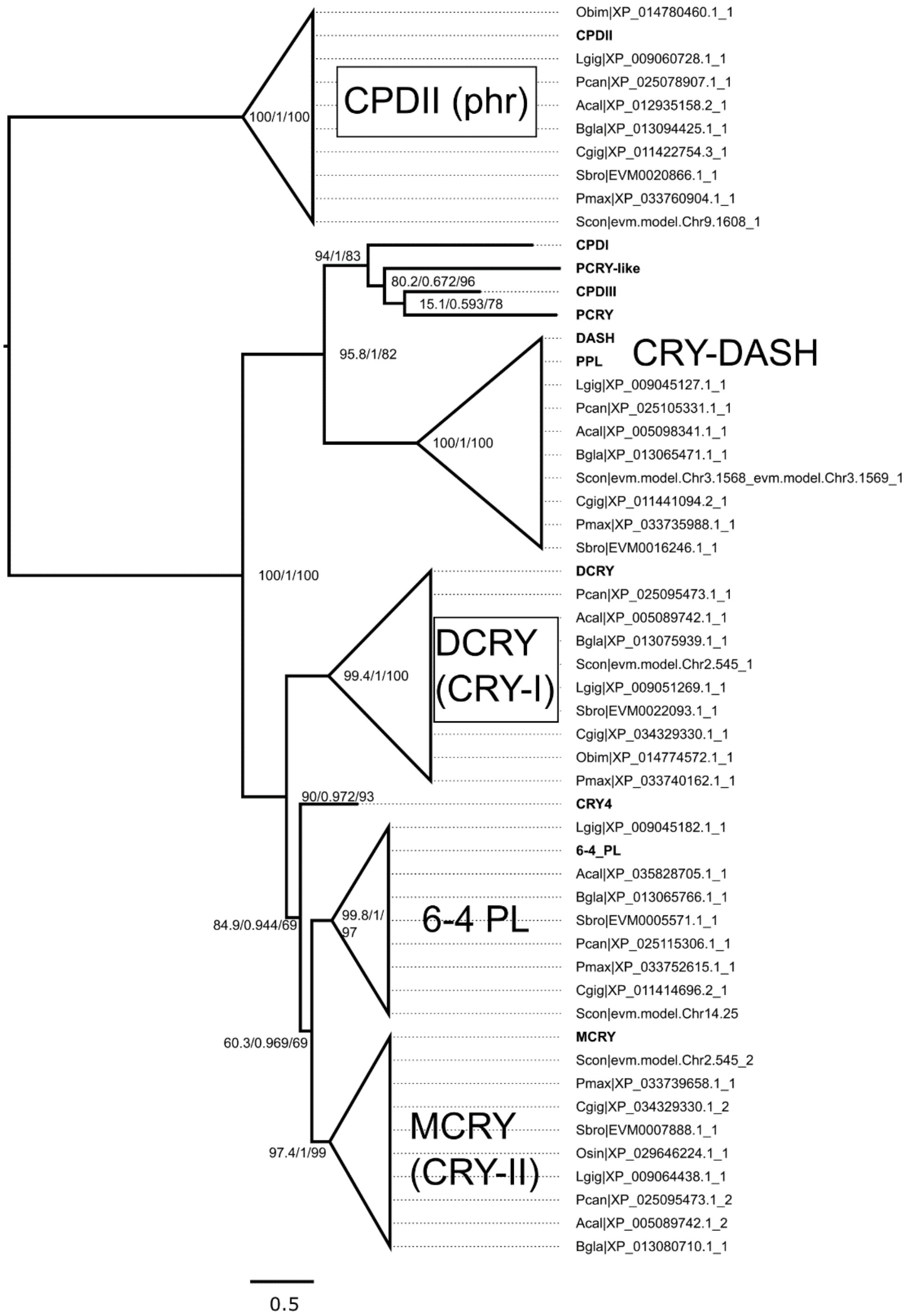
Obim|XP_014780460.1_1
CPDII
Lgig|XP_009060728.1_1
Pcan|XP_025078907.1_1
Acal|XP_012935158.2_1
Bgla|XP_013094425.1_1
Cgig|XP_011422754.3_1
Sbro|EVM0020866.1_1
Pmax|XP_033760904.1_1
Scon|evm.model.Chr9.1608_1
CPDII (phr)
100/1/100
CPDI
PCRY-like
CPDIII
PCRY
94/1/83
80.2/0.672/96
15.1/0.593/78
DASH
PPL
CRY-DASH
95.8/1/82
Lgig|XP_009045127.1_1
Pcan|XP_025105331.1_1
Acal|XP_005098341.1_1
Bgla|XP_013065471.1_1
Scon|evm.model.Chr3.1568_evm.model.Chr3.1569_1
Cgig|XP_011441094.2_1
Pmax|XP_033735988.1_1
Sbro|EVM0016246.1_1
100/1/100
100/1/100
DCRY
Pcan|XP_025095473.1_1
Acal|XP_005089742.1_1
Bgla|XP_013075939.1_1
Scon|evm.model.Chr2.545_1
Lgig|XP_009051269.1_1
Sbro|EVM0022093.1_1
Cgig|XP_034329330.1_1
Obim|XP_014774572.1_1
Pmax|XP_033740162.1_1
DCRY (CRY-I)
99.4/1/100
90/0.972/93
CRY4
Lgig|XP_009045182.1_1
6-4_PL
Acal|XP_035828705.1_1
Bgla|XP_013065766.1_1
Sbro|EVM0005571.1_1
Pcan|XP_025115306.1_1
Pmax|XP_033752615.1_1
Cgig|XP_011414696.2_1
Scon|evm.model.Chr14.25
6-4 PL
99.8/1/97
84.9/0.944/69
60.3/0.969/69
MCRY
Scon|evm.model.Chr2.545_2
Pmax|XP_033739658.1_1
Cgig|XP_034329330.1_2
Sbro|EVM0007888.1_1
Osin|XP_029646224.1_1
Lgig|XP_009064438.1_1
Pcan|XP_025095473.1_2
Acal|XP_005089742.1_2
Bgla|XP_013080710.1_1
MCRY (CRY-II)
97.4/1/99
0.5

Figure S5

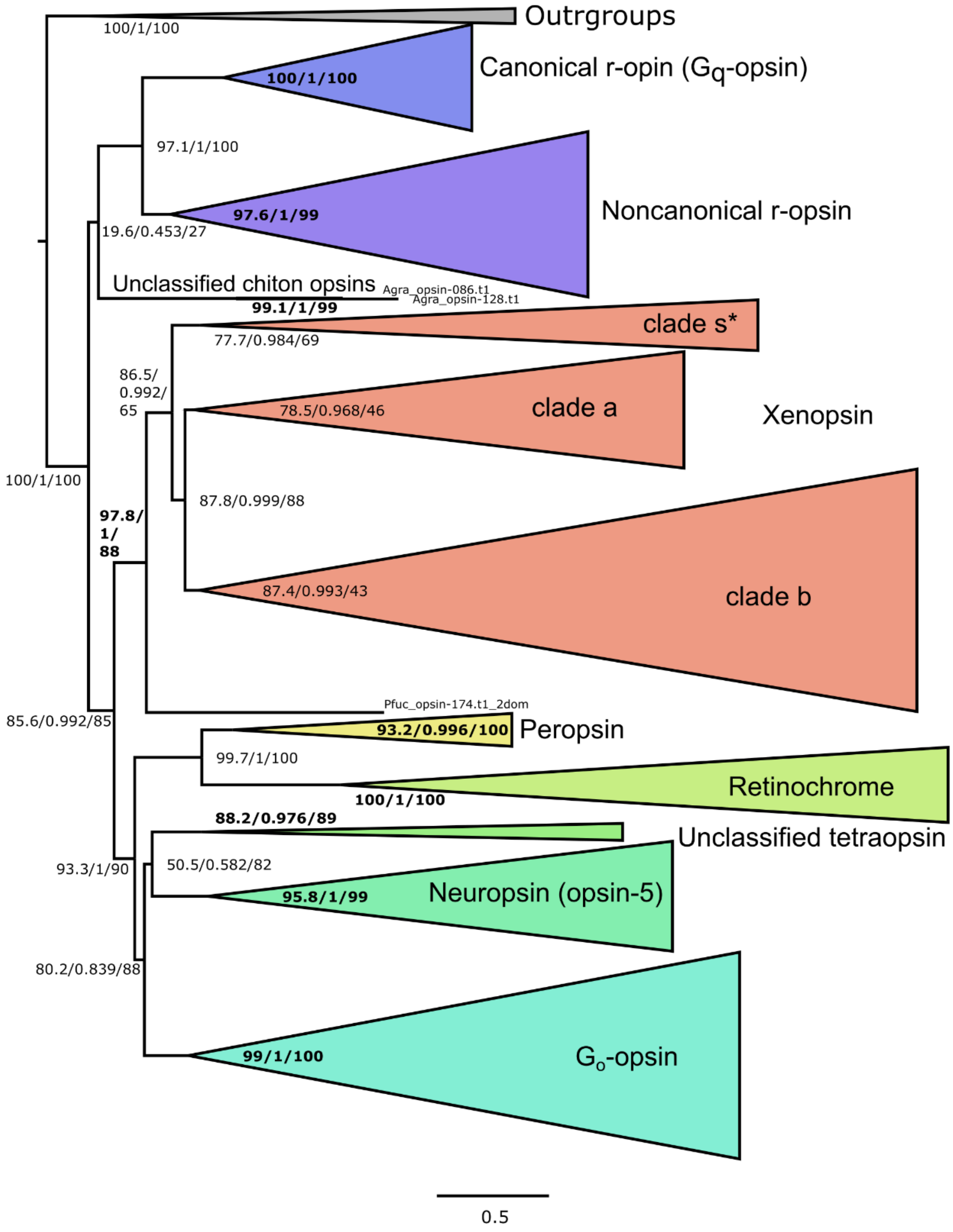
Outrgroups
100/1/100
Canonical r-opin (Gq-opsin)
100/1/100
97.1/1/100
Noncanonical r-opsin
97.6/1/99
19.6/0.453/27
Unclassified chiton opsins
Agra_opsin-086.t1
Agra_opsin-128.t1
99.1/1/99
clade s*
77.7/0.984/69
86.5/
0.992/
65
clade a
78.5/0.968/46
Xenopsin
100/1/100
87.8/0.999/88
97.8/
1/
88
clade b
87.4/0.993/43
Pfuc_opsin-174.t1_2dom
85.6/0.992/85
Peropsin
93.2/0.996/100
99.7/1/100
Retinochrome
100/1/100
88.2/0.976/89
Unclassified tetraopsin
93.3/1/90
50.5/0.582/82
Neuropsin (opsin-5)
95.8/1/99
80.2/0.839/88
Go-opsin
99/1/100
0.5

Figure S6

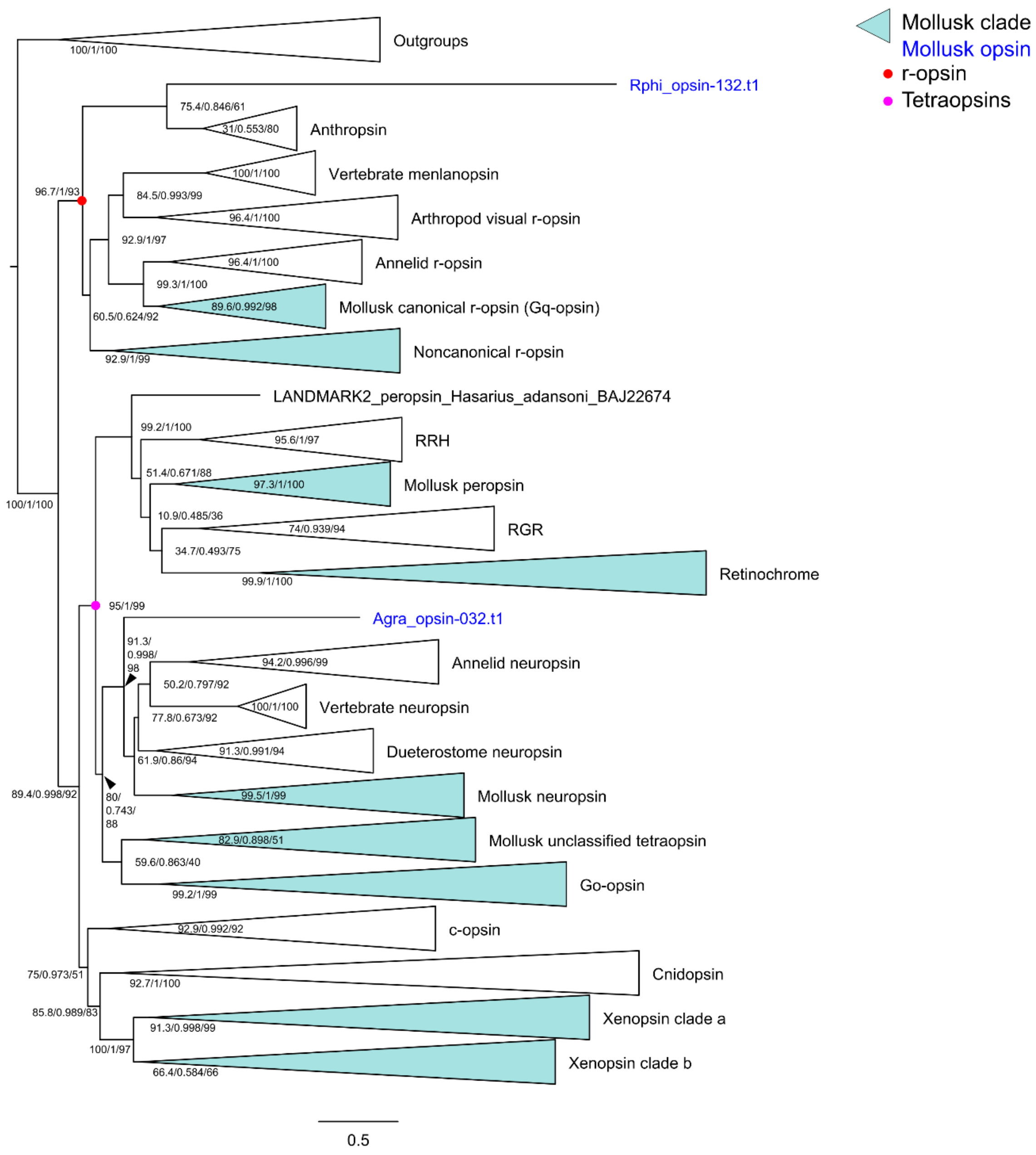

Mollusk clade
Mollusk opsin
r-opsin
Tetraopsins
Outgroups
100/1/100
Rphi_opsin-132.t1
75.4/0.846/61
31/0.553/80
Anthropsin
100/1/100
Vertebrate menlanopsin
96.7/1/93
84.5/0.993/99
96.4/1/100
Arthropod visual r-opsin
92.9/1/97
96.4/1/100
Annelid r-opsin
99.3/1/100
89.6/0.992/98
Mollusk canonical r-opsin (Gq-opsin)
60.5/0.624/92
92.9/1/99
Noncanonical r-opsin
LANDMARK2_peropsin_Hasarius_adansoni_BAJ22674
99.2/1/100
95.6/1/97
RRH
51.4/0.671/88
97.3/1/100
Mollusk peropsin
100/1/100
10.9/0.485/36
74/0.939/94
RGR
34.7/0.493/75
99.9/1/100
Retinochrome
95/1/99
Agra_opsin-032.t1
91.3/ 0.998/ 98
94.2/0.996/99
Annelid neuropsin
50.2/0.797/92
100/1/100
Vertebrate neuropsin
77.8/0.673/92
91.3/0.991/94
Dueterostome neuropsin
61.9/0.86/94
89.4/0.998/92
80/ 0.743/ 88
99.5/1/99
Mollusk neuropsin
82.9/0.898/51
Mollusk unclassified tetraopsin
59.6/0.863/40
99.2/1/99
Go-opsin
92.9/0.992/92
c-opsin
75/0.973/51
Cnidopsin
92.7/1/100
85.8/0.989/83
91.3/0.998/99
Xenopsin clade a
100/1/97
66.4/0.584/66
Xenopsin clade b
0.5

Figure S8

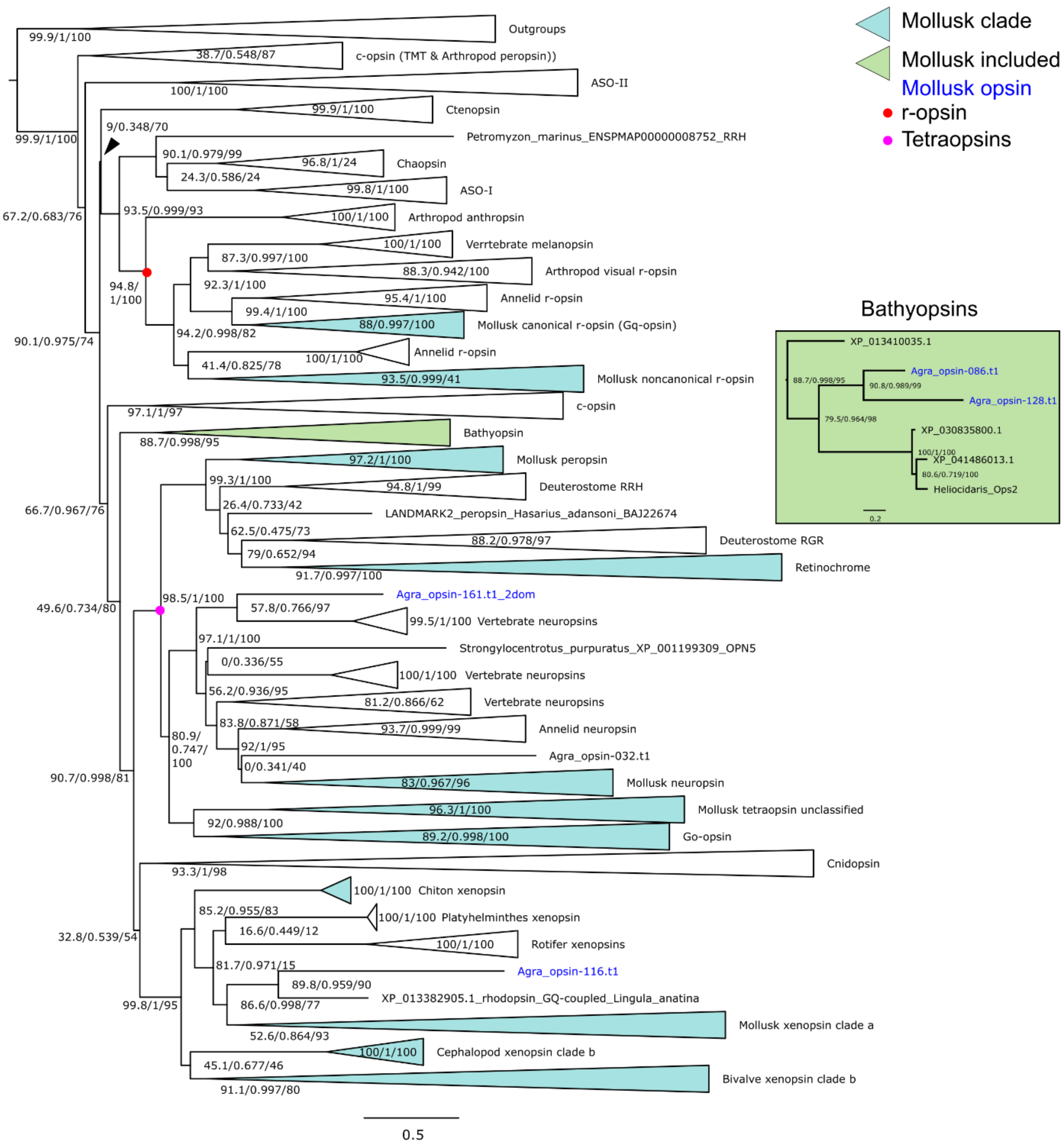
Mollusk clade
Mollusk included
Mollusk opsin
r-opsin
Tetraopsins
Outgroups
c-opsin (TMT & Arthropod peropsin))
ASO-II
Ctenopsin
Petromyzon_marinus_ENSPMAP00000008752_RRH
Chaopsin
ASO-I
Arthropod anthropsin
Verrtebrate melanopsin
Arthropod visual r-opsin
Annelid r-opsin
Mollusk canonical r-opsin (Gq-opsin)
Annelid r-opsin
Mollusk noncanonical r-opsin
c-opsin
Bathyopsin
Mollusk peropsin
Deuterostome RRH
LANDMARK2_peropsin_Hasarius_adansoni_BAJ22674
Deuterostome RGR
Retinochrome
Agra_opsin-161.t1_2dom
99.5/1/100 Vertebrate neuropsins
Strongylocentrotus_purpuratus_XP_001199309_OPN5
100/1/100 Vertebrate neuropsins
Vertebrate neuropsins
Annelid neuropsin
Agra_opsin-032.t1
Mollusk neuropsin
Mollusk tetraopsin unclassified
Go-opsin
Cnidopsin
100/1/100 Chiton xenopsin
100/1/100 Platyhelminthes xenopsin
Rotifer xenopsins
Agra_opsin-116.t1
XP_013382905.1_rhodopsin_GQ-coupled_Lingula_anatina
Mollusk xenopsin clade a
Cephalopod xenopsin clade b
Bivalve xenopsin clade b
99.9/1/100
38.7/0.548/87
100/1/100
99.9/1/100
9/0.348/70
99.9/1/100
90.1/0.979/99
96.8/1/24
24.3/0.586/24
99.8/1/100
67.2/0.683/76
93.5/0.999/93
100/1/100
100/1/100
87.3/0.997/100
88.3/0.942/100
92.3/1/100
95.4/1/100
94.8/
1/100
99.4/1/100
88/0.997/100
94.2/0.998/82
90.1/0.975/74
100/1/100
41.4/0.825/78
93.5/0.999/41
97.1/1/97
88.7/0.998/95
97.2/1/100
99.3/1/100
94.8/1/99
66.7/0.967/76
26.4/0.733/42
62.5/0.475/73
88.2/0.978/97
79/0.652/94
91.7/0.997/100
98.5/1/100
49.6/0.734/80
57.8/0.766/97
97.1/1/100
0/0.336/55
56.2/0.936/95
81.2/0.866/62
83.8/0.871/58
93.7/0.999/99
80.9/
0.747/
100
92/1/95
0/0.341/40
90.7/0.998/81
83/0.967/96
96.3/1/100
92/0.988/100
89.2/0.998/100
93.3/1/98
85.2/0.955/83
16.6/0.449/12
100/1/100
32.8/0.539/54
81.7/0.971/15
89.8/0.959/90
99.8/1/95
86.6/0.998/77
52.6/0.864/93
100/1/100
45.1/0.677/46
91.1/0.997/80
0.5
Bathyopsins
XP_013410035.1
Agra_opsin-086.t1
88.7/0.998/95
90.8/0.989/99
Agra_opsin-128.t1
79.5/0.964/98
XP_030835800.1
100/1/100
XP_041486013.1
80.6/0.719/100
Heliocidaris_Ops2
0.2

Figure S9

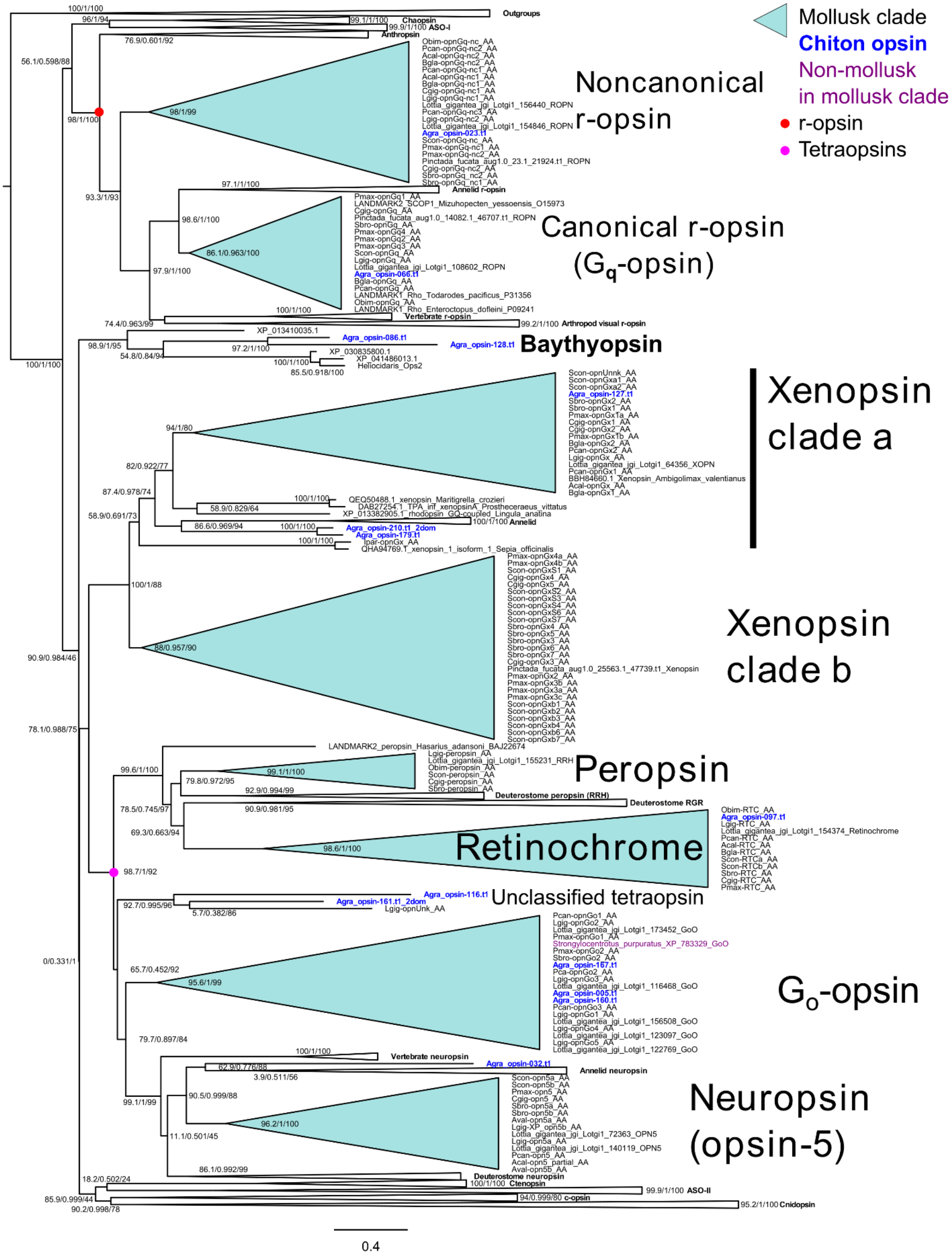

Mollusk clade
Chiton opsin
Non-mollusk in mollusk clade
r-opsin
Tetraopsins
Outgroups
Chaopsin
ASO-I
Anthropsin
Noncanonical r-opsin
Annelid r-opsin
Canonical r-opsin (G_q-opsin)
Vertebrate r-opsin
Arthropod visual r-opsin
Baythyopsin
Xenopsin clade a
Xenopsin clade b
Peropsin
Deuterostome peropsin (RRH)
Deuterostome RGR
Retinochrome
Unclassified tetraopsin
G_o-opsin
Vertebrate neuropsin
Annelid neuropsin
Neuropsin (opsin-5)
Deuterostome neuropsin
Ctenopsin
ASO-II
c-opsin
Cnidopsin
0.4

Figure S12

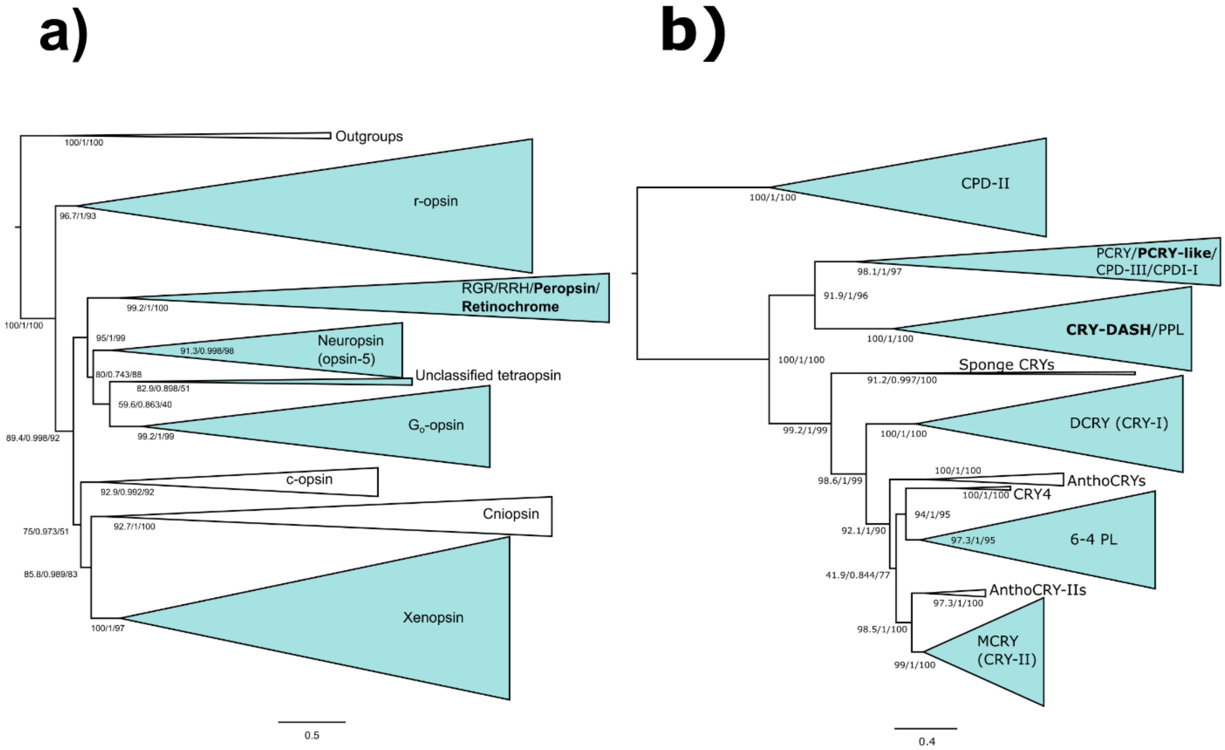
a)
Outgroups
100/1/100
r-opsin
96.7/1/93
RGR/RRH/Peropsin/
Retinochrome
99.2/1/100
100/1/100
95/1/99
91.3/0.998/98
Neuropsin
(opsin-5)
Unclassified tetraopsin
80/0.743/88
82.9/0.898/51
59.6/0.863/40
G_o-opsin
99.2/1/99
89.4/0.998/92
c-opsin
92.9/0.992/92
Cniopsin
92.7/1/100
75/0.973/51
85.8/0.989/83
Xenopsin
100/1/97
0.5
b)
CPD-II
100/1/100
PCRY/PCRY-like/
CPD-III/CPDI-I
98.1/1/97
91.9/1/96
CRY-DASH/PPL
100/1/100
100/1/100
Sponge CRYs
91.2/0.997/100
DCRY (CRY-I)
99.2/1/99
100/1/100
100/1/100
AnthoCRYs
98.6/1/99
100/1/100 CRY4
94/1/95
92.1/1/90
97.3/1/95
6-4 PL
41.9/0.844/77
AnthoCRY-IIs
97.3/1/100
98.5/1/100
MCRY
(CRY-II)
99/1/100
0.4
