# Supplementary figures and images for: Molluscan Genomes Reveal Extensive Differences in Photopigment Evolution Across the Phylum
Source: Mol Biol Evol. 2023 Dec 1;40(12):msad263. doi: 10.1093/molbev/msad263 (PMC10733189; doi:10.1093/molbev/msad263)

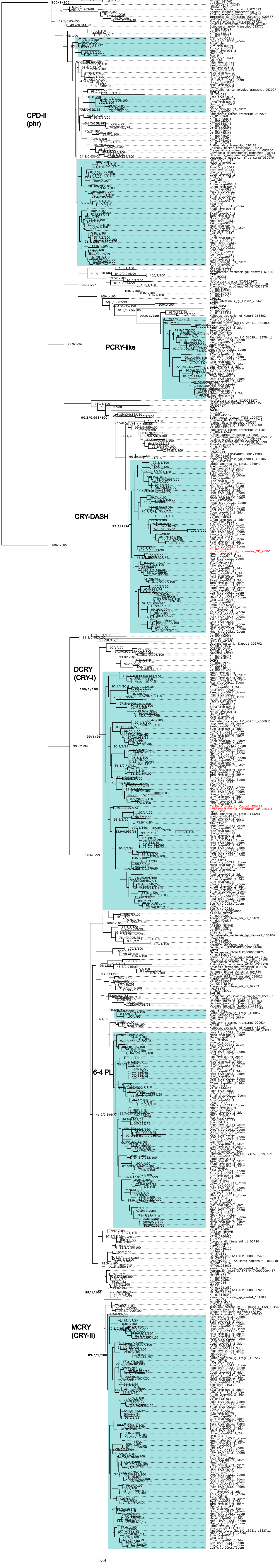

Supplement: msad263_Supplementary_Data [file msad263_supplementary_data.zip › Figure_S11.pdf]
