# Supplementary material for: Molluscan Genomes Reveal Extensive Differences in Photopigment Evolution Across the Phylum
Source: Mol Biol Evol. 2023 Dec 1;40(12):msad263. doi: 10.1093/molbev/msad263 (PMC10733189; doi:10.1093/molbev/msad263)
Supplement: msad263_Supplementary_Data [file Figure_S7.pdf › Figure_S7.pdf]

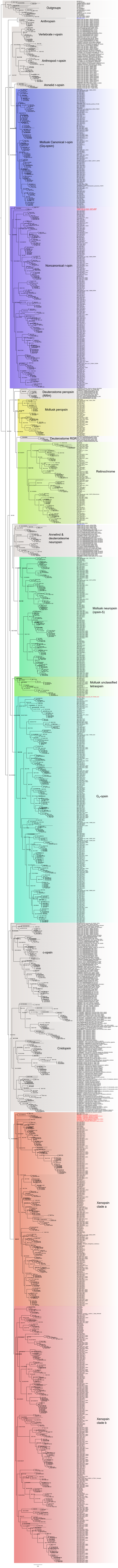
Outgroups
Anthropsin
Vertebrate r-opsin
Arthropod r-opsin
Annelid r-opsin
Mollusk Canonical r-opin (Gq-opsin)
Noncanonical r-opin
Deuterostome peropsin (RRH)
Mollusk peropsin
Deuterostome RGR
Retinochrome
Annelid & deuterostome neuropsin
Mollusk neuropsin (opsin-5)
Mollusk unclassified tetraopsin
Go-opsin
c-opsin
Cnidopsin
Xenopsin clade a
Xenopsin clade b
